# Supplementary material for: Rapid lightsheet fluorescence imaging of whole Drosophila brains at nanoscale resolution by potassium acrylate-based expansion microscopy
Source: Nat Commun. 2024 Dec 30;15:10911. doi: 10.1038/s41467-024-55305-8 (PMC11685761; doi:10.1038/s41467-024-55305-8)
Supplement: Supplementary file 1 — Supplementary Information [file 41467_2024_55305_MOESM1_ESM.pdf]

Supplementary Information

Rapid lightsheet fluorescence imaging of whole *Drosophila* brains at nanoscale resolution by potassium acrylate-based expansion microscopy

Authors: Xuejiao Tian[1,2,3#], Tzu-Yang Lin[4#], Po-Ting Lin[1], Min-Ju Tsai[1], Hsin Chen[1], Wen-Jie Chen[5,6], Chia-Ming Lee[1], Chiao-Hui Tu[1], Jui-Cheng Hsu[1], Tung-Han Hsieh[1], Yi-Chung Tung[1], Chien-Kai Wang[7], Suewei Lin[6], Li-An Chu[8], Fan-Gang Tseng[1,2,3], Yi-Ping Hsueh[6], Chi-Hon Lee[4], Peilin Chen[1], Bi-Chang Chen[1,4]*

Affiliations:

1. Research Center for Applied Sciences, Academia Sinica, Taipei 11529, Taiwan
2. Nano Science and Technology Program, Taiwan International Graduate Program, Academia Sinica 11529, Taiwan
3. Department of Engineering and System Science, National Tsing Hua University, Hsinchu 300, Taiwan
4. Institute of Cellular and Organismic Biology, Academia Sinica, Taipei 11529, Taiwan
5. Taiwan International Graduate Program in Interdisciplinary Neuroscience, National Cheng Kung University and Academia Sinica, Taipei 11529, Taiwan
6. Institute of Molecular Biology, Academia Sinica, Taipei, 11529, Taiwan
7. Department of Mechanical Engineering, National Taiwan University, Taipei, 106319, Taiwan
8. Department of Biomedical Engineering and Environmental Sciences, National Tsing Hua University, Hsinchu, Taiwan

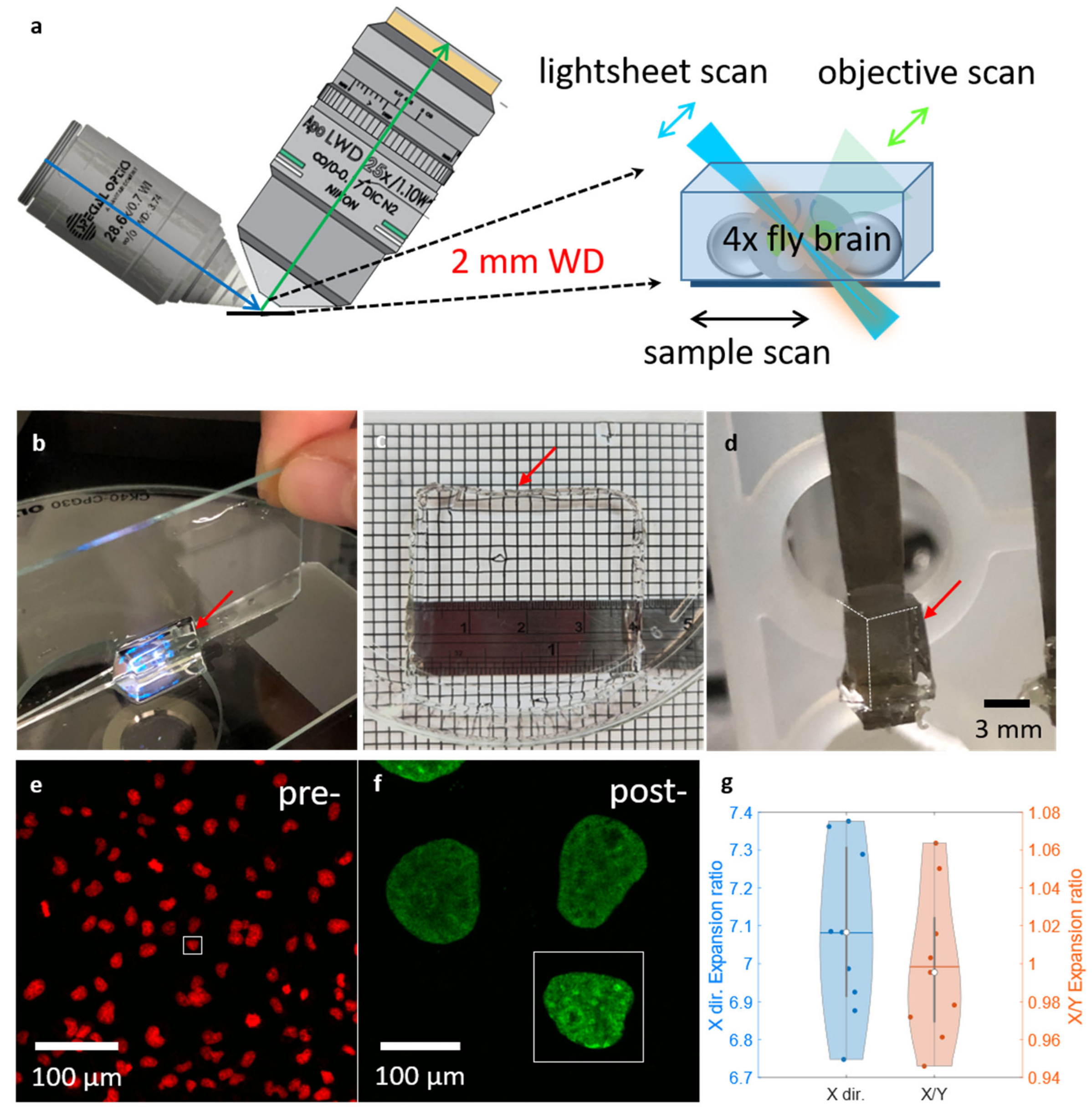

Supplementary Figure 1. ExLLSM scanning scheme and hydrogel for X10 ExM vs *in situ* KA-ExM **(a)** ExLLSM's imaging core composed of an excitation objective (Special Optics, 0.65 NA, 3.74 mm WD) and a detection objective (Nikon, CFI Apo LWD 25XW, 1.1 NA, 2 mm WD) whose focal plane is co-incident with the lightsheet. Zoomed-in view for the 4x expansion fly brain embedded inside the gel and scanning by either moving the lightsheet and detection objective together (ligthsheet scan mode) or translating the specimen with a piezo stage through the stationary lightsheet with tilted angle with respect to the objectives **(b)** Original 10x ExM gel with water texture as marked by red arrow, not rigid enough for vertical mounting. For demonstration, a glass slide used to hold it still. **(c)** An *in situ* KA-ExM hydrogel with 4-cm size, the gel doesn't

have watery property since a clear cut as marked by the red arrow **(d)** An *in situ* KA expanded sample with 6 mm x 4 mm x 3 mm mounted in vertical orientation on the L shape holder, and the white dashed lines used to linemate the shape of this transparent hydrogel pointed by the red arrow. **(e)** and **(f)** Images of *in situ* KA-ExM cultured COS7 cells pre- and post-expansion. The cell marked by a white square is the same cell before and after expansion. Note that the same scale bars are in (e) and (f). **(g)** A violin plot for 9 measurements on the identical cells for expansion ratio calculation for *x* and *y* directions and the laterally isotropic expansion. Note that three coverslips hosting cultured cells were analyzed separately, with three cells chosen from each coverslip for pre- and post-expansion size measurements. Source data are provided as a Source Data file.

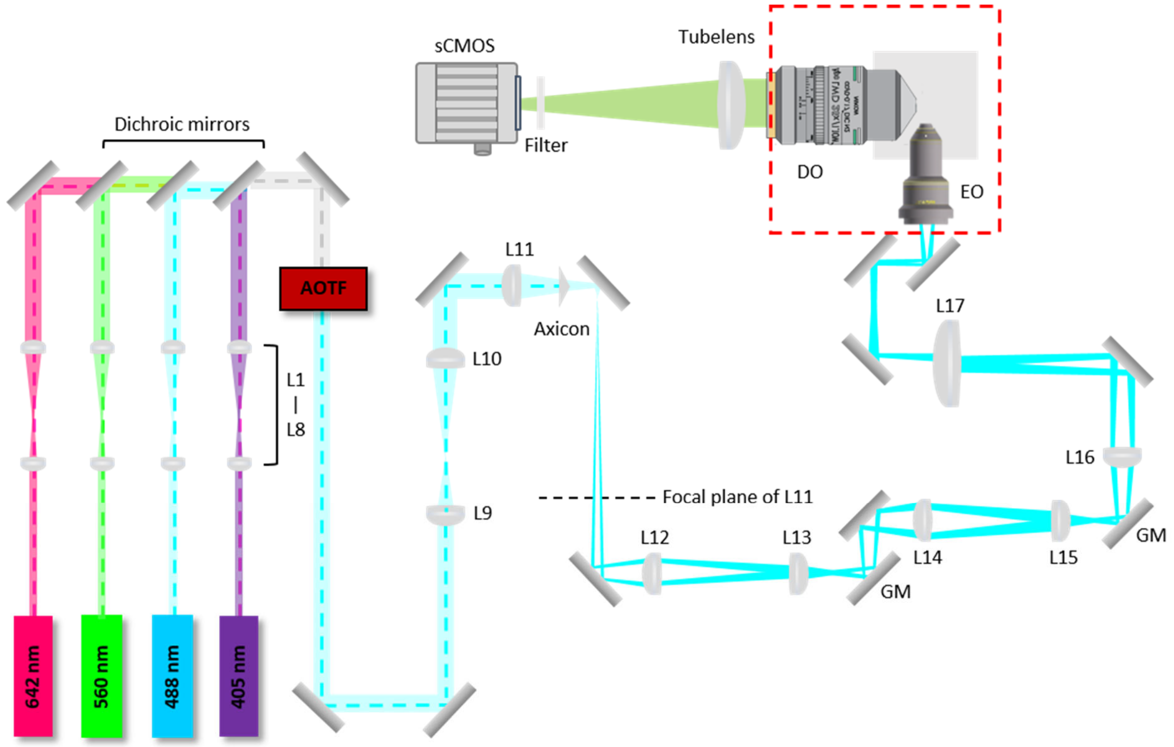

Supplementary Figure 2. The schematic of the optical system for axicon-based Bessel lightsheet microscope (ΔBLX). The beam from a laser combiner equipped with 405 nm, 488 nm, 560 nm and 642 nm lasers. The lasers were combined with long-pass dichroic filters and aligned collinearly before entering an acousto-optical tunable filter by two lenses (8 mm FL and 20 mm FL) (L1~L8). L9: 60 mm FL lens; L10: 200 mm FL lens; L11: 250 mm FL; L12: 100 mm FL; L13: 125 mm FL GM: galvo mirror; L14: 75 mm FL; L15: 75 mm FL; L16: 150 mm FL; L17: 400 mm FL; EO: excitation objective; DO: detection objective.

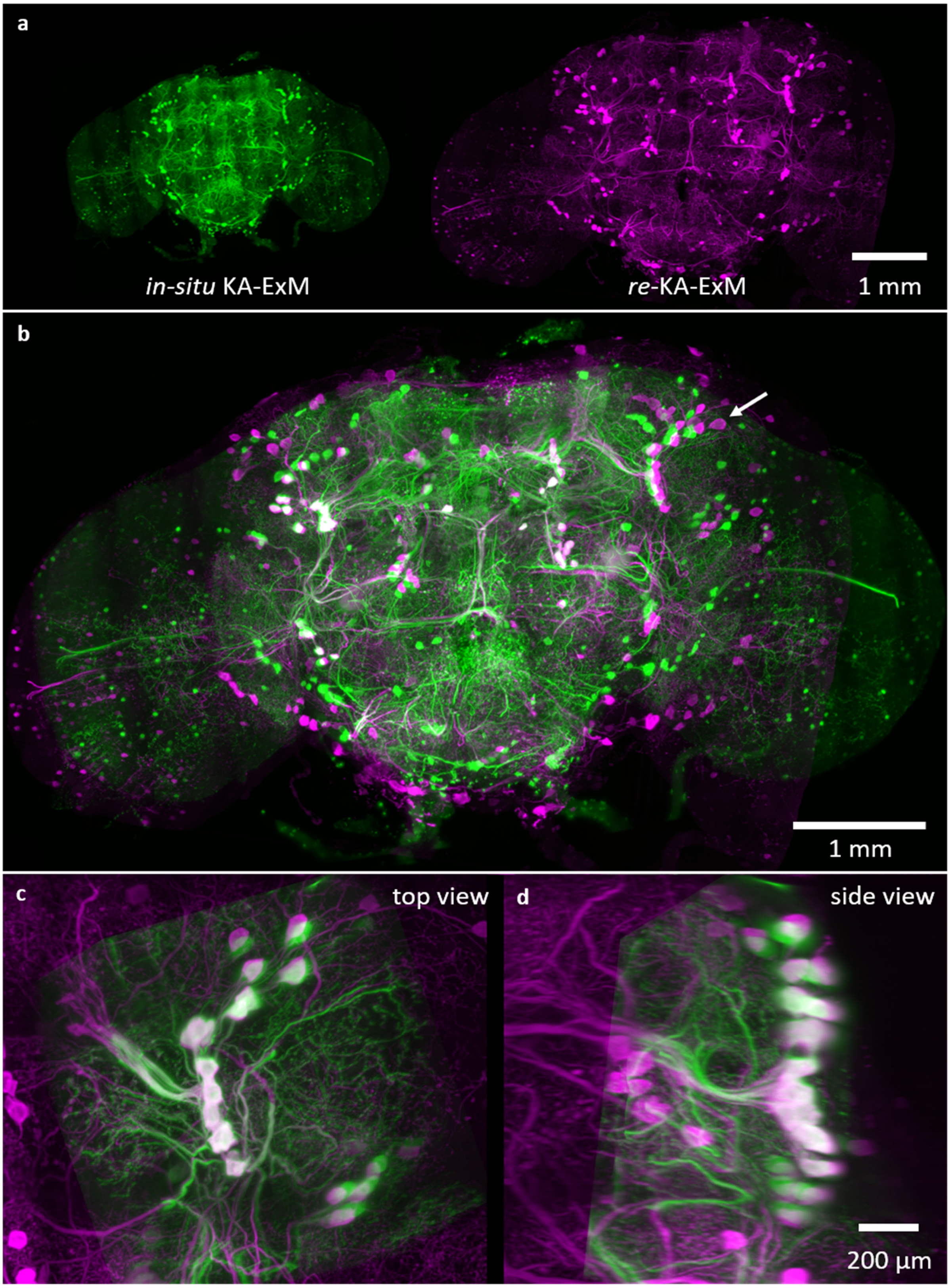
a
in-situ KA-ExM
re-KA-ExM
1 mm
b
1 mm
c
top view
d
side view
200 µm

Supplementary Figure 3. The comparison of *in situ* KA-ExM (*8x*) and *re*-KA-ExM (*13x*) at high spatial resolution in 3D on the identical TH-GAL4, 20XUAS-6XGFP/+ fly brain in maximum intensity projection images from ΔBLX. **(a)**The green color for *in situ* KA-ExM whole fly brain images and the magenta for *re*-KA-ExM on the green one where the scale bar 1 mm. **(b)** The registration for the images in (a) by multiplying an expansion factor of 1.5 on the green color fly brain in x, y, and z direction. **(c)** the top view of the enlarged area indicated by white arrow in (b), showing the cell bodies retained after 8x and 13x expansion. **(d)** The side view of (c) in the axial direction, showing the non-isotropic deformation for the iterative process on the *in situ* KA-ExM but the content is maintained. The scale bar is 200 μm.

a

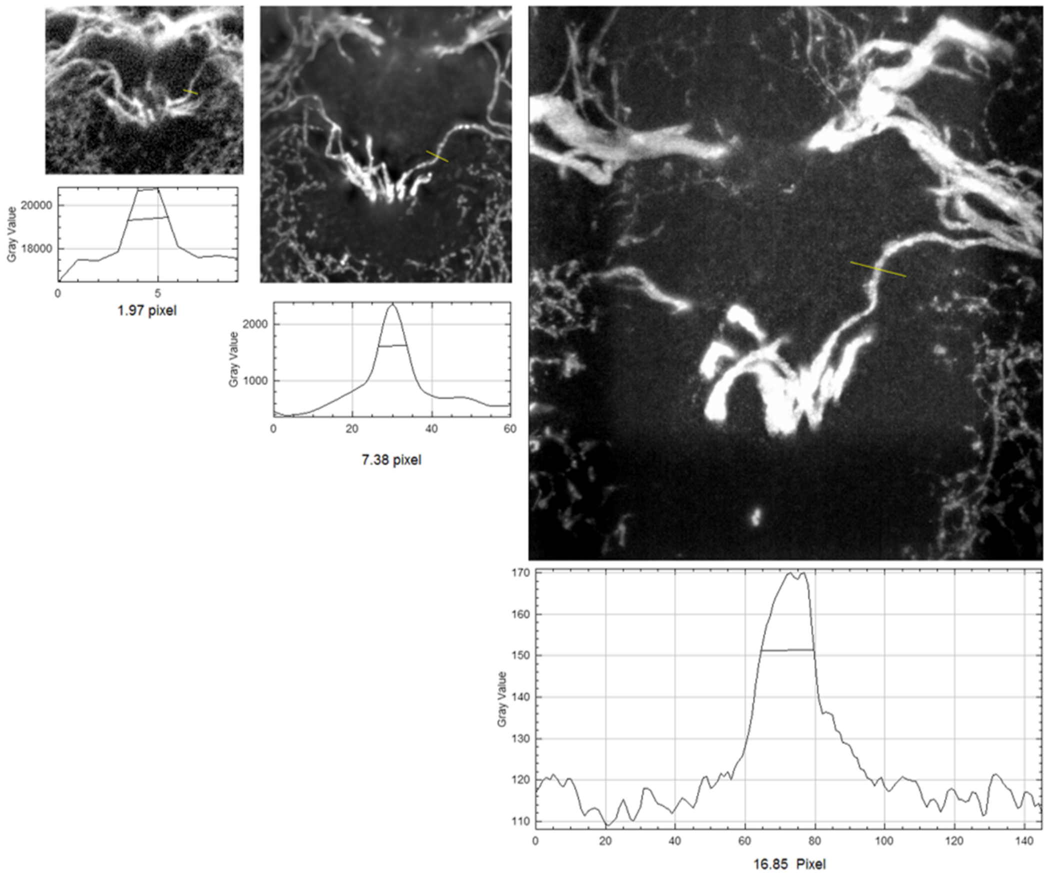

Gray Value
20000
18000
0
5
1.97 pixel
Gray Value
2000
1000
0
20
40
60
7.38 pixel
Gray Value
170
160
150
140
130
120
110
0
20
40
60
80
100
120
140
16.85 Pixel

b

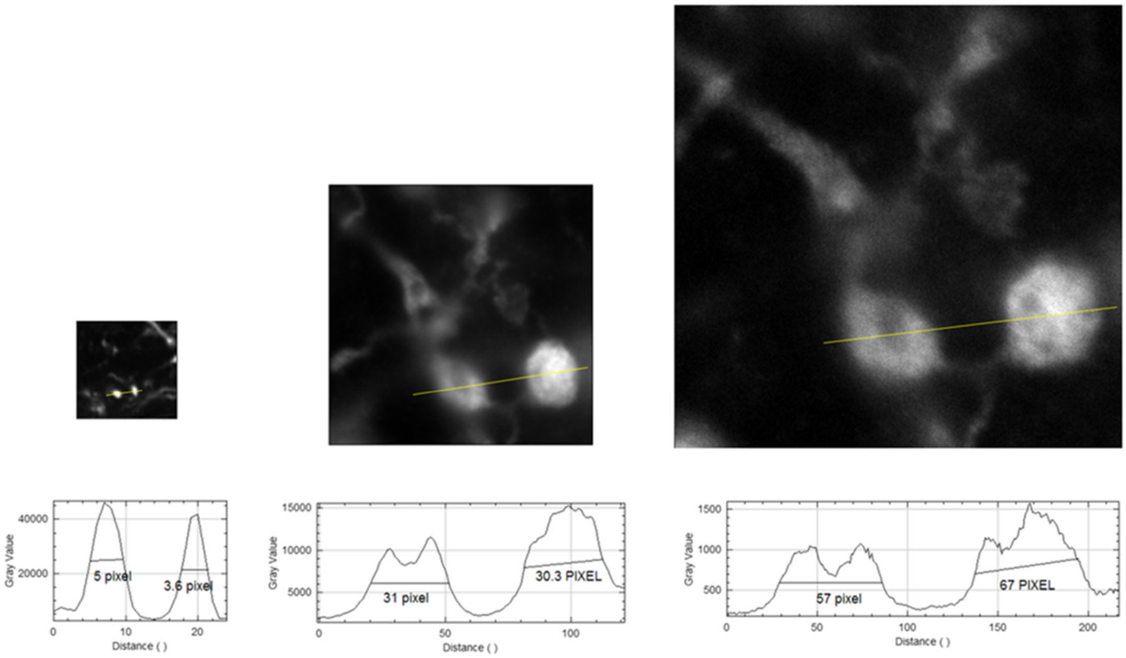

Gray Value
40000
20000
5 pixel
3.6 pixel
0
10
20
Distance ( )
Gray Value
15000
10000
5000
31 pixel
30.3 PIXEL
0
50
100
Distance ( )
Gray Value
1500
1000
500
57 pixel
67 PIXEL
0
50
100
150
200
Distance ( )

Supplementary Figure 4. Characterization of *in situ* KA-ExM (*8x*) and *re*-KA-ExM (*13x*) at high spatial resolution of the TH-GAL4, 20XUAS-6XGFP/+ fly brain. **(a)** Line profile along a neuron fiber in a similar area of the EB. The full width at half maximum measurements for the (cleared) original, *in situ* KA-ExM, *re*-KA-ExM samples are 1.97, 7.38 and 16.85 pixels, respectively. **(b)** Line profile along two presynaptic boutons in the PRW region. The full width at half maximum measurements for the (cleared) original, *in situ* KA-ExM, and *re*-KA-ExM samples are shown. Note that the original image was acquired from a cleared fly brain in order to obtain a high resolution. The fly brains are already slightly swollen due to being subjected to the clearing reagent (CUBIC).

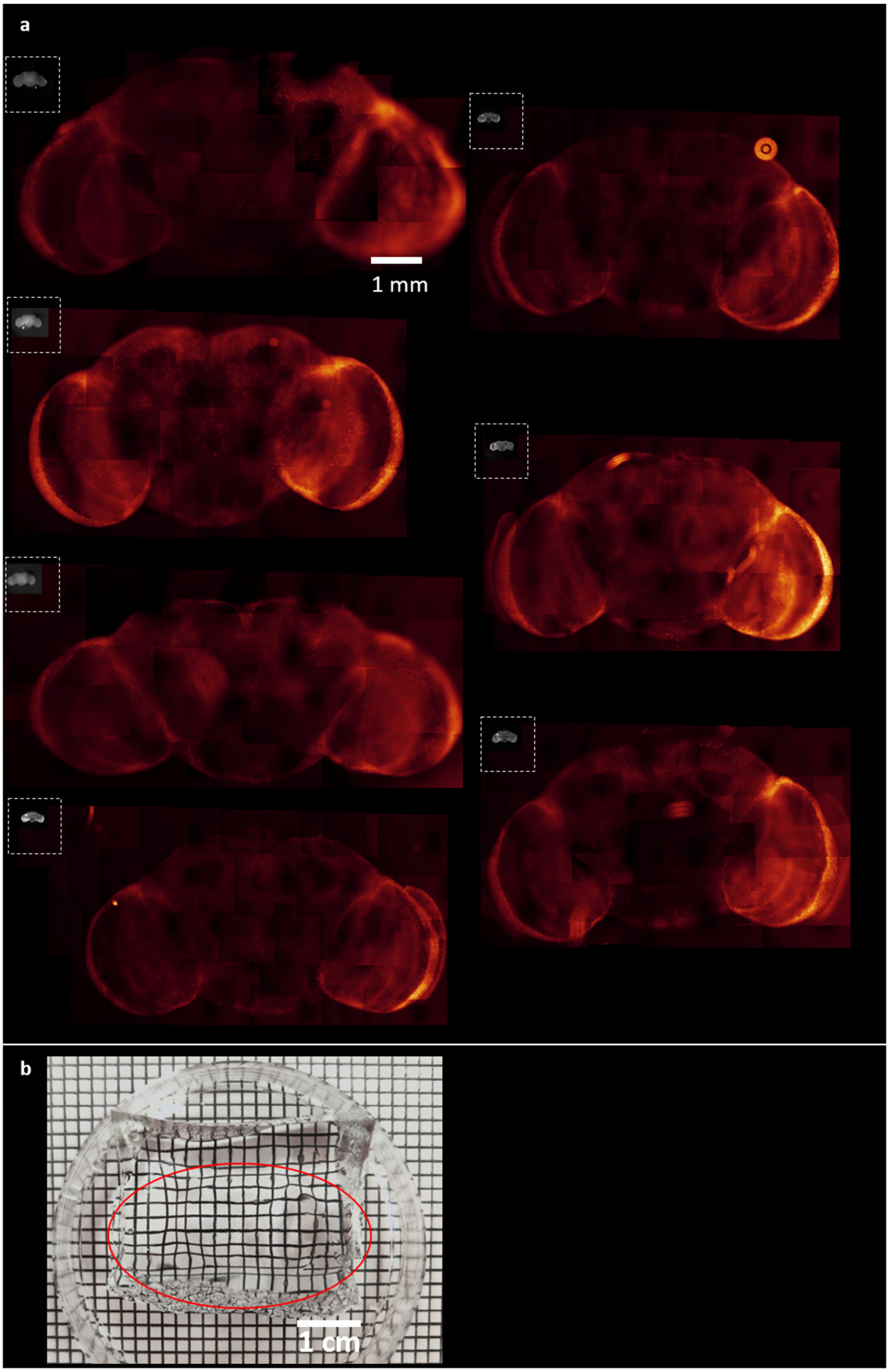
a
1 mm
b
1 cm

Supplementary Figure 5. The epi-fluorescence imaging for pre- and post-PKA-ExM on *Drosophila* brain for 7 trials shown in **(a)** where the original fly brains shown in grey color and dashed box and expanded fly brain shown in the glow color. **(b)** one representative 2nd hydrogel from *re*-PKA-ExM, in which the expanded fly brain is outlined in red (size > 2 cm), the grid size 2.5 mm. The measurements of lateral expansion ratio calculated in the **Supplementary Table 4** resulted in ~15X expansion ratio for PKA-ExM. Expansion ratio in long axis of fly brain is 14.2 ± 0.98; expansion ratio in short axis is 15.4 ± 1.91 based on 7 measurements. 2-dimensional distortion in **(a)** was calculated by comparing the size of the same fly brain along two directions before and after expansion resulted in 0.93 ± 0.11. Source data are provided as a Source Data file.

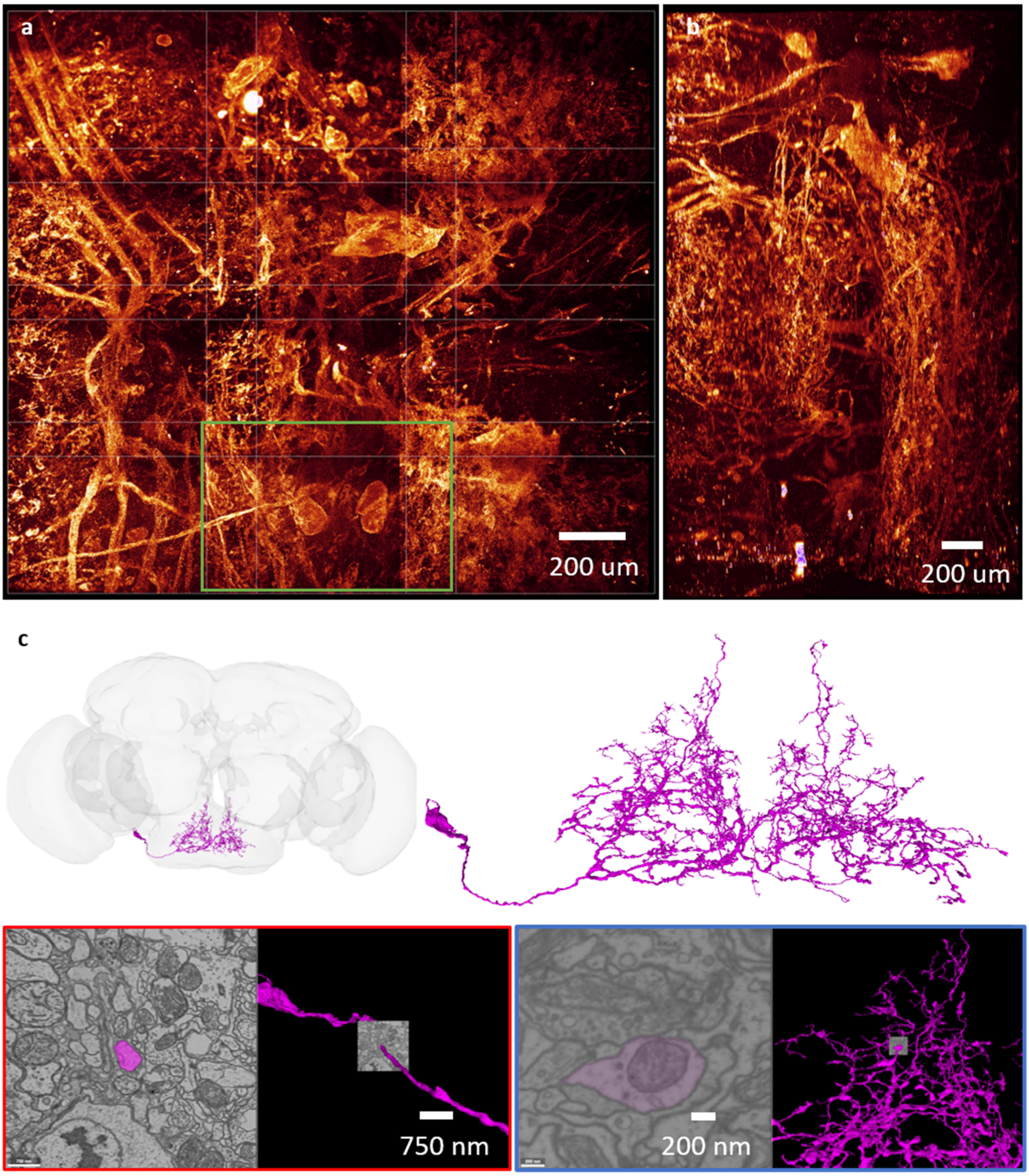

Supplementary Figure 6. The re-PKA-ExM on TH-GAL4, 20XUAS-6XGFP/+ fly brain in 3D in maximum intensity projection images from ΔBLX and FlyWire resource. **(a)** 12 tiles of the PKA-ExM in glow color for dopaminergic neurons near central complex acquired by ΔBLX. Scale bar 200 µm. **(b)** The side view of the green tile in (a) showing the axial resolution is improved by expansion microscopy even with NA=0.6 objective lens detection. **(c)** PRW.55 dopaminergic neurons in the FlyWire EM resource, showing the diameter of the fine process and mitochondria inside the synapse structures under EM.

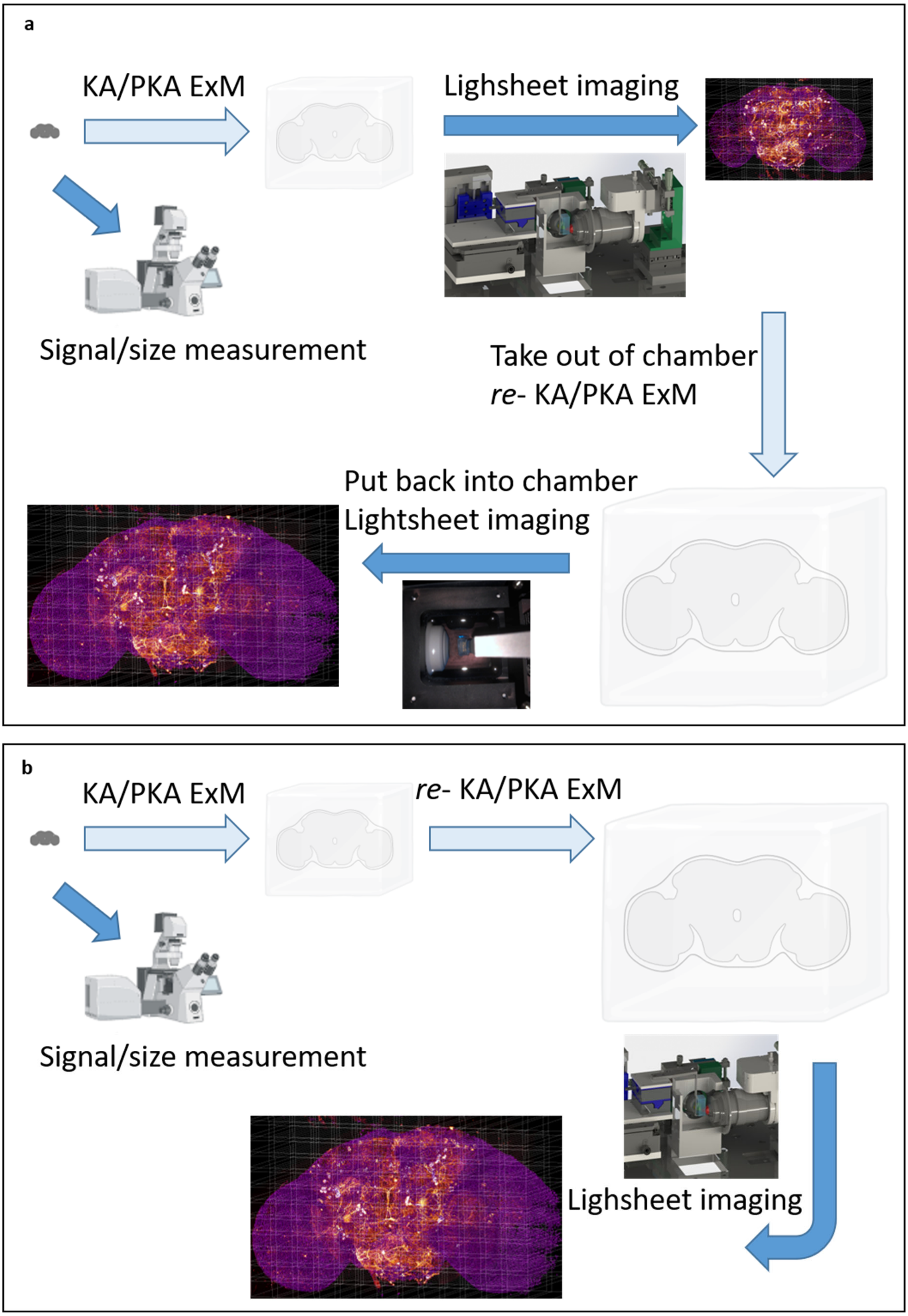
a
KA/PKA ExM
Lighsheet imaging
Signal/size measurement
Take out of chamber
re- KA/PKA ExM
Put back into chamber
Lightsheet imaging
b
KA/PKA ExM
re- KA/PKA ExM
Signal/size measurement
Lighsheet imaging

Supplementary Figure 7. Characterization of iterative ExM on potassium (poly)acrylate-based hydrogels for step-by-step *vs* end-product imaging. **(a)** The original fly brain is examined by epi-fluorescence microscopy and measured. Following KA/PKA ExM, the hydrogels are mounted and transferred to the ΔBLX chamber for 3D whole brain imaging. After lightsheet imaging, the hydrogel is removed from the water chamber and subjected to *re*-KA/PKA ExM. The *re*-KA/PKA hydrogel is placed back into the ΔBLX chamber and undergoes lightsheet whole brain imaging for characterization. **(b)** The original fly brain is examined by epi-fluorescence microscopy and measured. Following KA/PKA and *re*-KA/PKA protocols, the hydrogel is mounted and transferred into the ΔBLX chamber for characterization by lightsheet whole brain imaging. Note that the hydrogel may suffer sample shrinkage due to the mounting glue or from long-term imaging during the first examination under the lightsheet microscope. Figure icons created with BioRender.com.

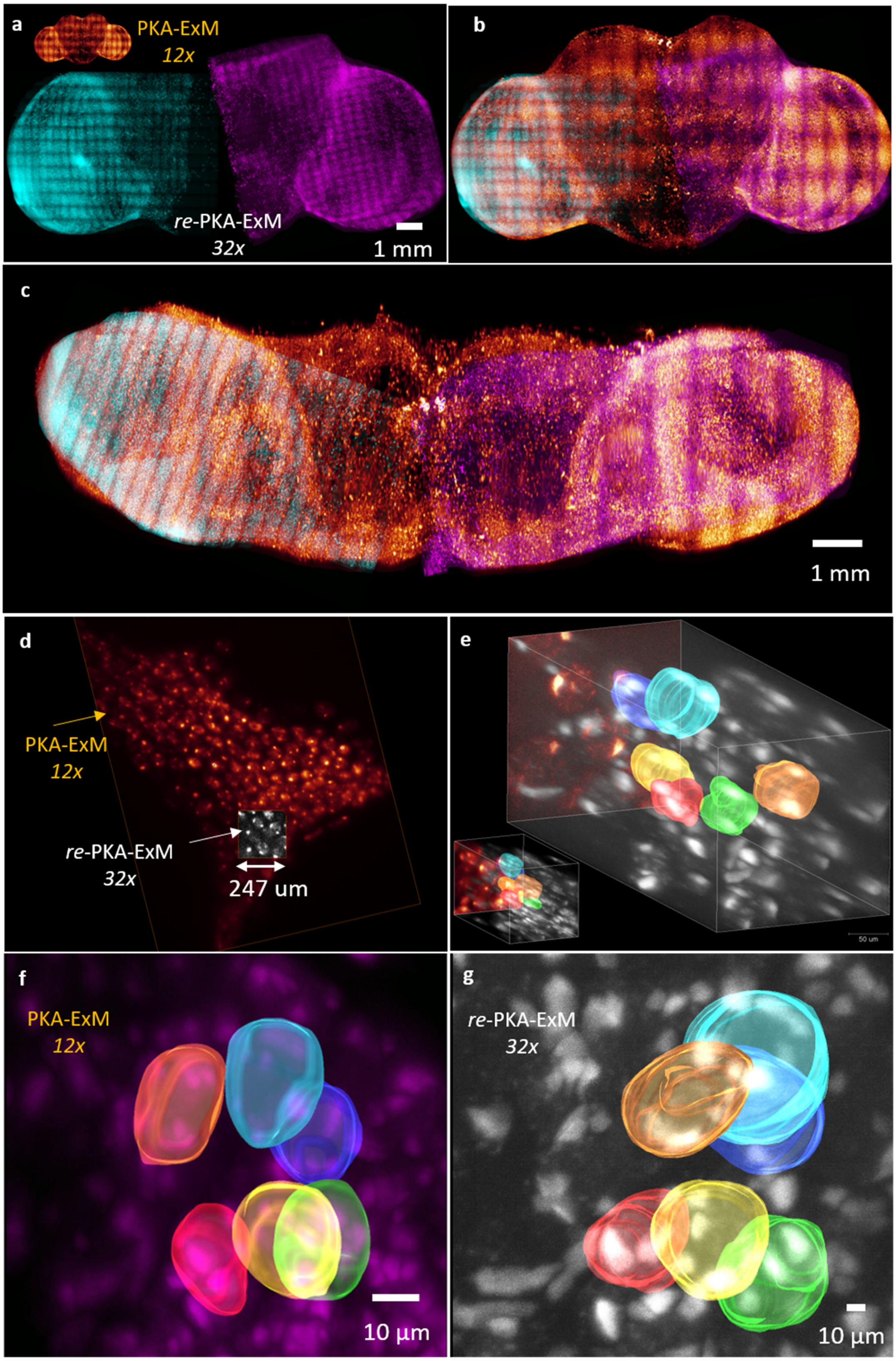

a
PKA-ExM
12x
re-PKA-ExM
32x
1 mm
b
c
1 mm
d
PKA-ExM
12x
re-PKA-ExM
32x
247 um
e
f
PKA-ExM
12x
10 µm
g
re-PKA-ExM
32x
10 µm

Supplementary Figure 8. The comparison of PKA-ExM (12x) and *re*-PKA-ExM (32x) at high spatial resolution in 3D on the identical fly brain with DAPI-nuclei stained in maximum intensity projection images from ΔBLX. **(a)** PKA-ExM in glow color and re-PKA-ExM in cyan or magenta colors. Note that the scale bar 1 mm. **(b)** The registration for the 12x images in (a) by multiplying scaling factor of 2.9, 2.6 and 2.6 in *x,y,z* directions, separately, to match the iterative expansion process resulted in 19.1x9.6x5.7 mm^3. **(c)** the side view of (b). Note that the grid pattern coming from the lightsheet imaging stitching. Each grid made of 2048x1408x z slices, z varying on the sample thickness and scanning step size. **(d)** The overlapped slice view of PKA-ExM (12x) and *re*-PKA ExM (32x) for the same selected area in the optic lobe, where the PKA ExM grow image multiplied the scale factors of 2.9, 3.0, and 4.1 separately in *x,y,z* directions to match *re*-PKA-ExM grey raw image in 3D with 15-degree rotation. **(e)** 3D renderings of the same selected regions in the grey area in (d), representing PKA-ExM (left) and *re*-PKA-ExM (right). In both cases, six nuclei have been segmented and rendered in different colors within the 3D image and xy view shown in **(f)** PKA-ExM and **(g)** *re*-PKA-ExM.

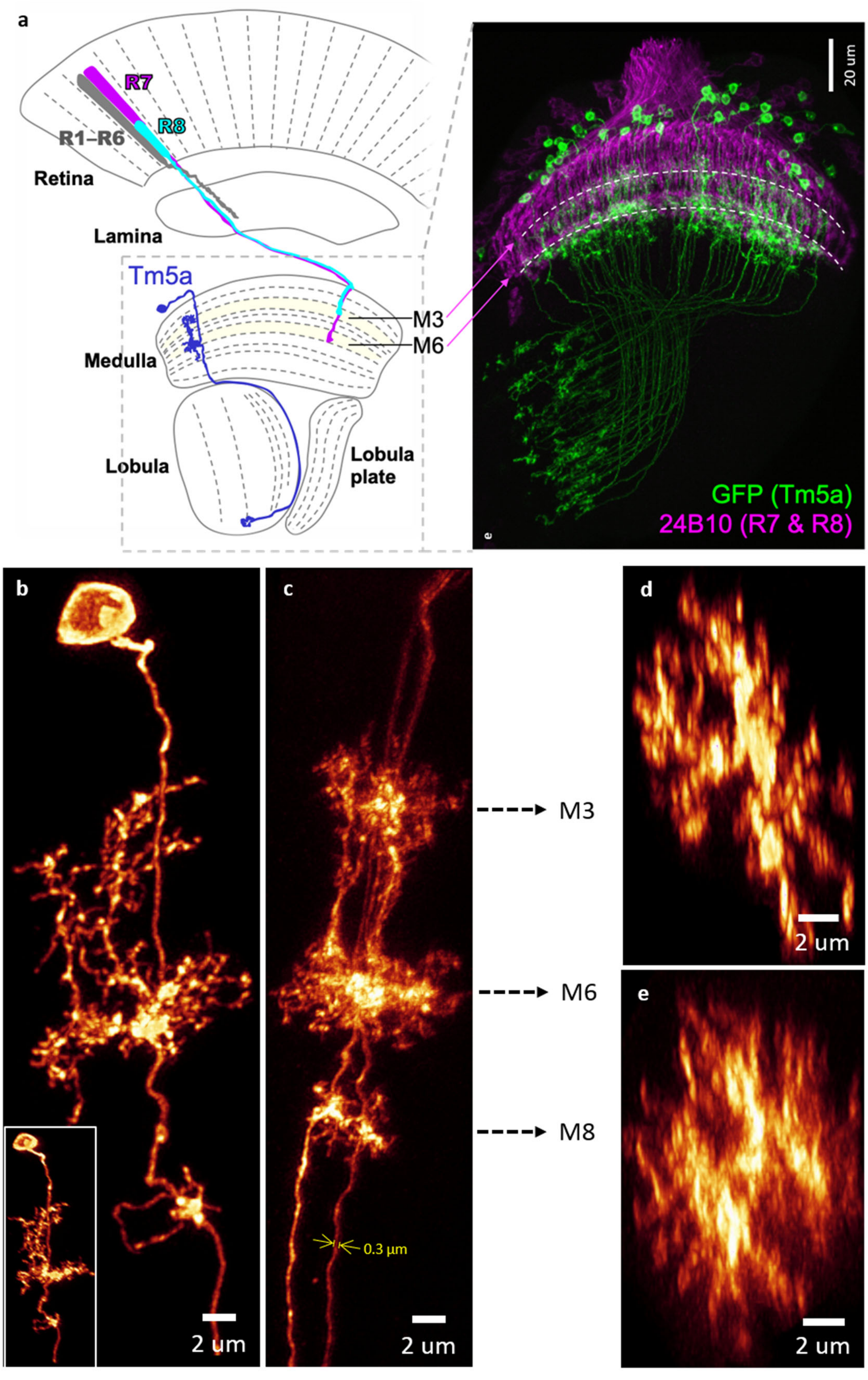

a
R7
R8
R1–R6
Retina
Lamina
Tm5a
Medulla
M3
M6
Lobula
Lobula plate
20 um
GFP (Tm5a)
24B10 (R7 & R8)
b
2 um
c
0.3 μm
2 um
M3
M6
M8
d
2 um
e
2 um

Supplementary Figure 9. The confocal images of Tm5a neuron in the optic lobe acquired in Zeiss LSM 880 with 20x NA=1.1 objective lens. **(a)** Schematic illustration of the retina, lamina, and medulla structures in the *Drosophila* optic lobe. The transmedullary neuron Tm5a (blue) extends its dendrites predominantly into the M3 layer (R8 axon terminal, cyan), M6 layer (R7 axon terminal, magenta), and M8 layer. Tm5a neurons were analyzed in flies expressing Tm5a split-LexA > CD4tdGFP. The GFP signal (green) was detected using an anti-GFP antibody. Photoreceptors R7 and R8 were visualized using 24B10 antibody (magenta). Scale bar: 20 µm. **(b)** A single Tm5a neuron and tilted view in the inset **(c)** two adjacent Tm5a neurons spanning lateral branches extend to reach neighboring columns in M3, M6, and M8 layers. **(d)** and **(e)** the top views for the dendrite field in M6 layer in (b) and (c) separately.

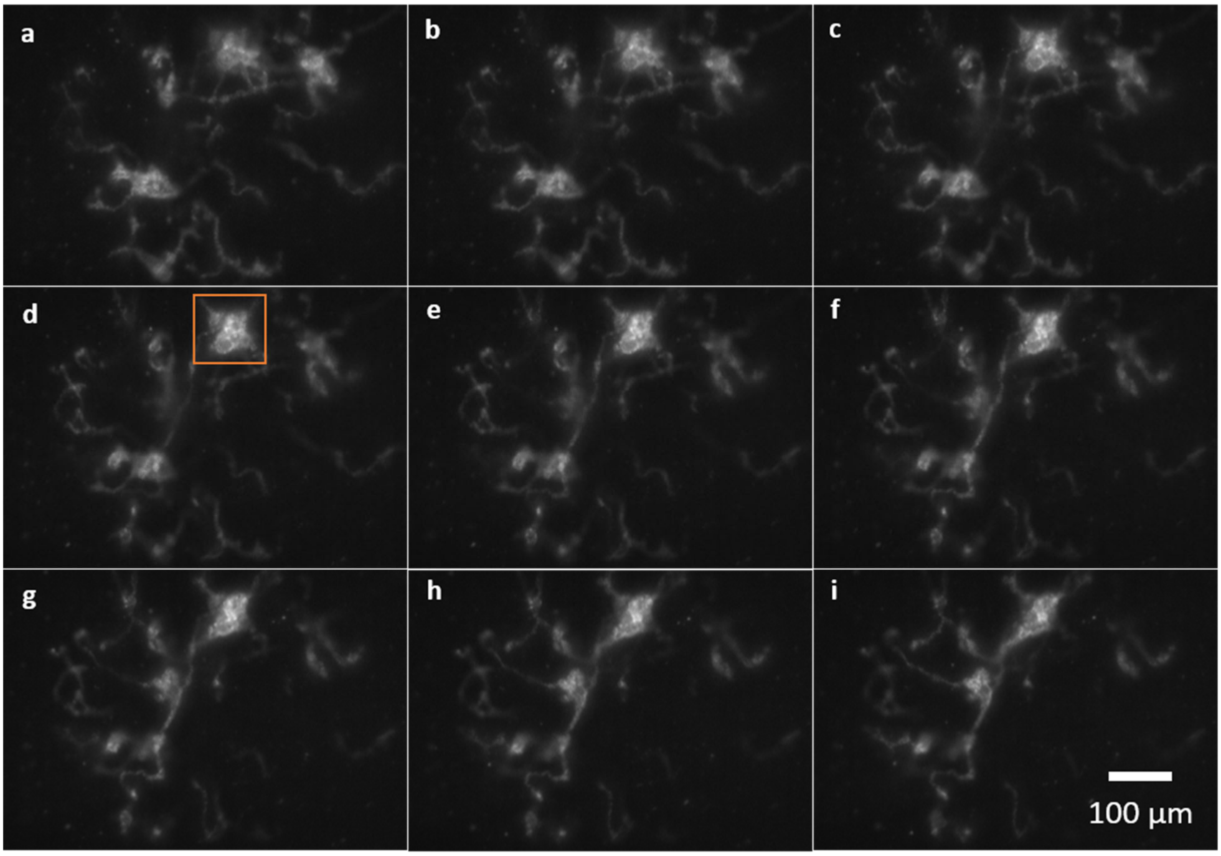

Supplementary Figure 10. The raw images of re-PKA-ExM fly optic lobe in the orange inset of Figure 5d, where Tm5a neurons with the dendritic processes. **(a)**~**(i)** the sliced views at the z interval of 6 μm. Note that the orange box in **(d)**, the same area shown in the inset of Figure 5d.

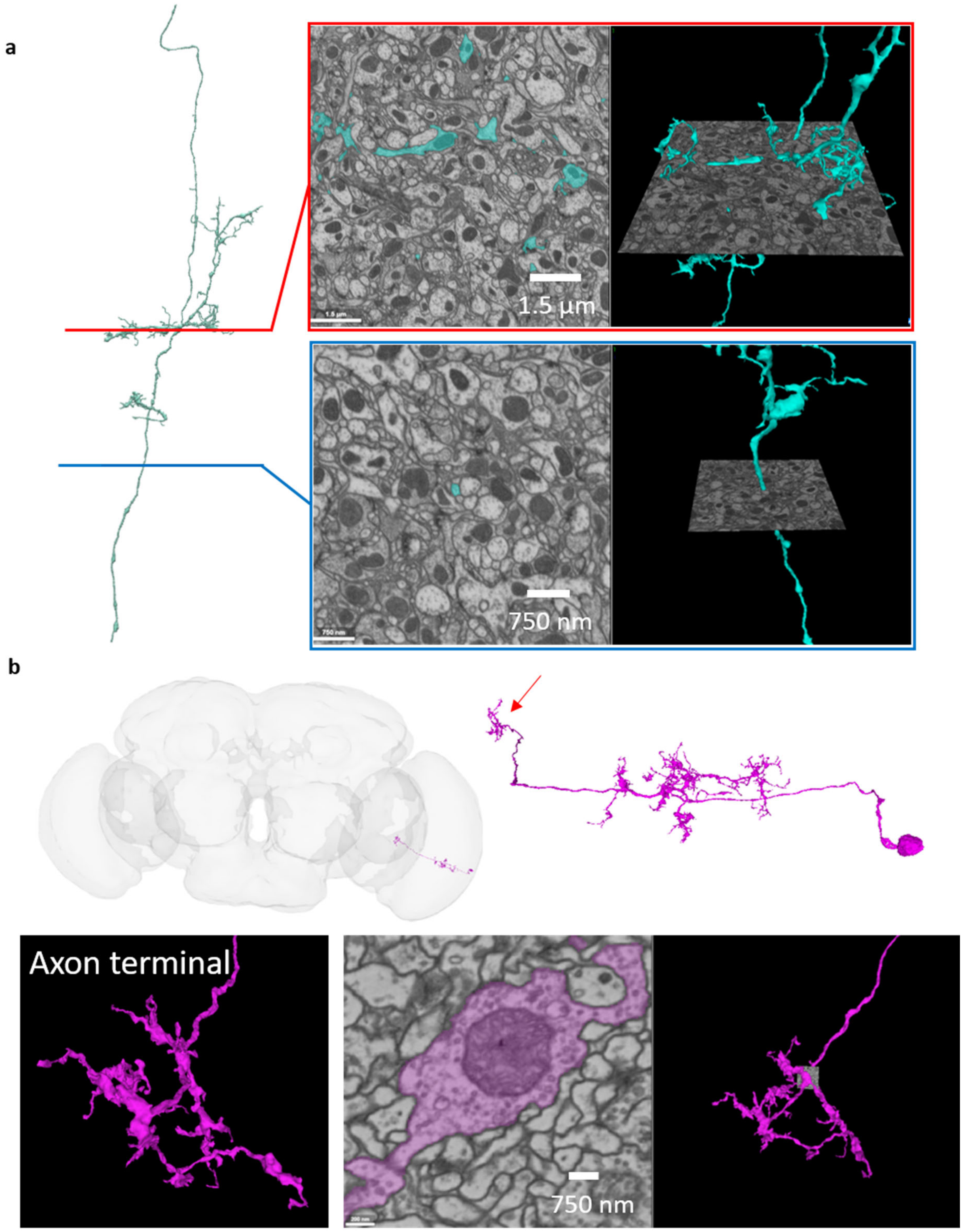
a
1.5 µm
1.5 µm
750 nm
750 nm
b
Axon terminal
750 nm

Supplementary Figure 11. The Tm5a visual neuron EM data from NeuPrint and FlyWire resources. **(a)** Tm5a neuron EM data from NeuPrint and sliced view at M6 and M8 showing the size of the axon fibers and complexity of the branches at M6. **(b)** Tm5a neuron (ME.LO.2431) from FlyWire. Zoomed at Axon terminal as indicated by red arrow for the 3D and slice views.

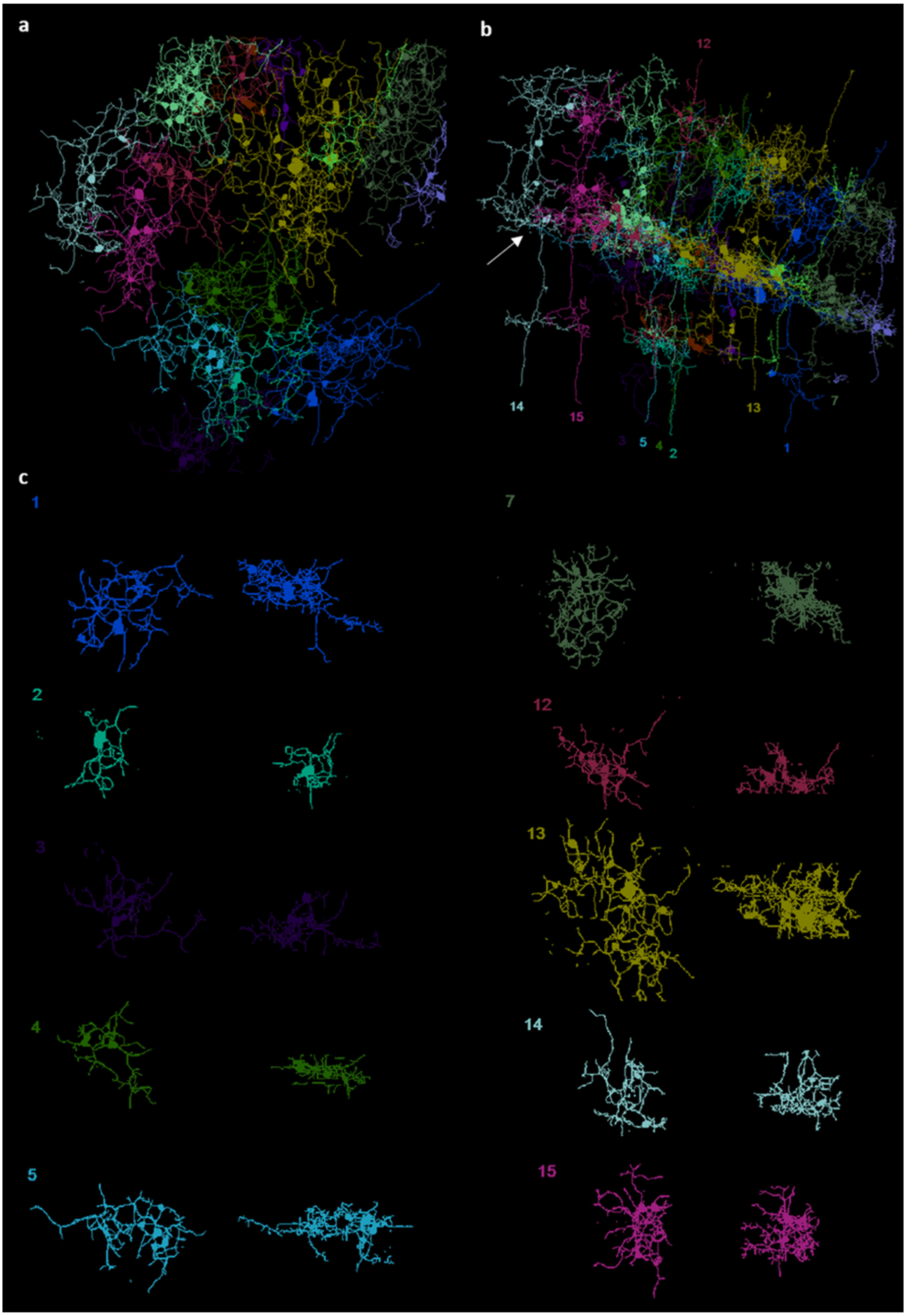

a
b
12
14
15
3
5
4
2
13
1
7
c
1
7
2
12
3
13
4
14
5
15

Supplementary Figure 12. The skeletonized Tm5a neurons in the *re*-PKA ExM fly optic lobe shown in the white dashed box in Figure 5b. **(a)** and **(b)** The top view and side view of a group of skeletonized Tm5a neurons in M3, M6 and M8 layers. **(c)** The top view (left) and side view (right) of the individual and colored Tm5a neurons with dendritic processes in M6 layer marked with white arrowhead in **(b)**.

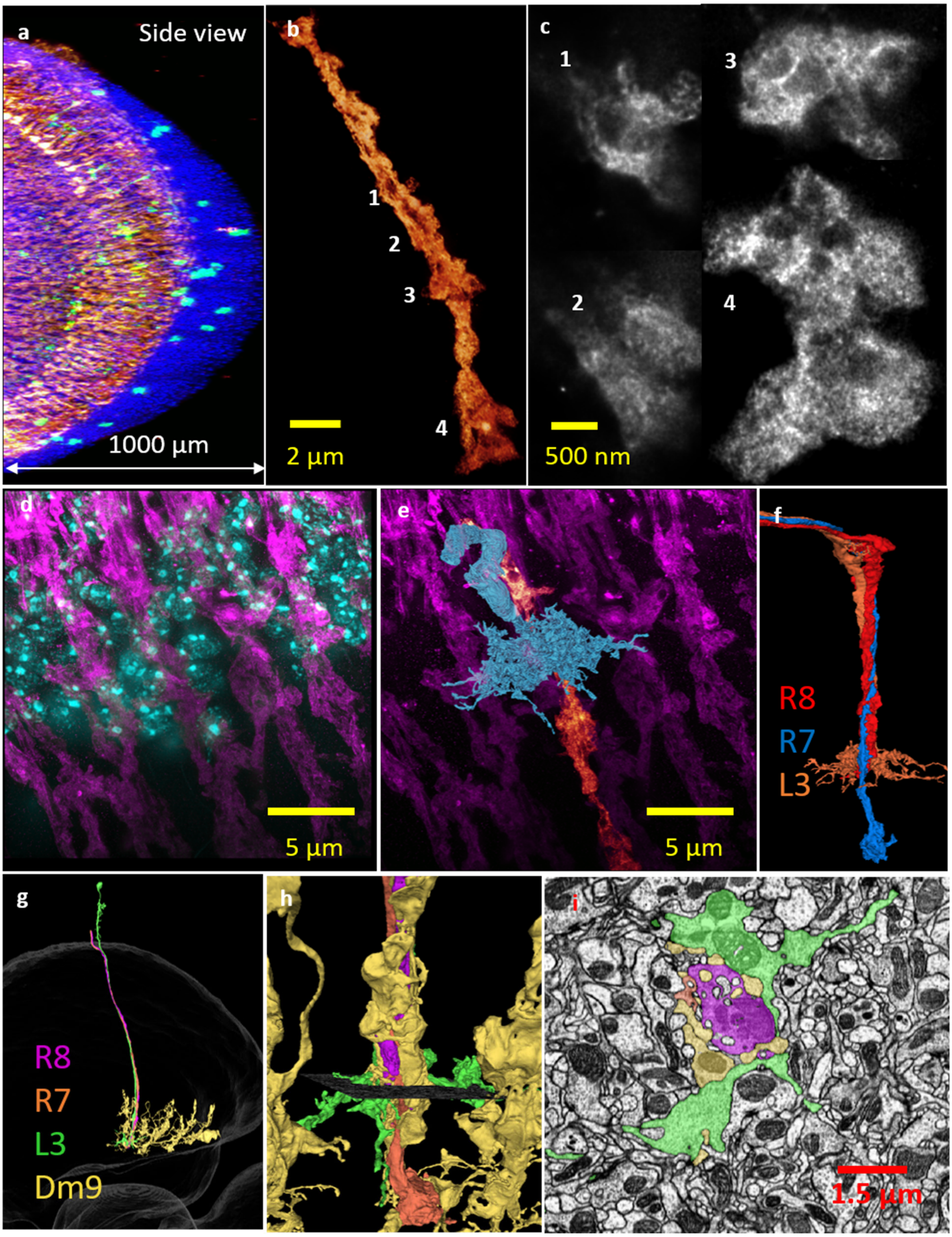

a
Side view
1000 µm
b
1
2
3
4
2 µm
c
1
2
3
4
500 nm
d
5 µm
e
5 µm
f
R8
R7
L3
g
R8
R7
L3
Dm9
h
i
1.5 µm

Supplementary Figure 13. Multicolor 3D KA-ExM lightsheet imaging of L3 single cell MARCM clone generated by *Ort*$^{C1\text{-}3}$*-GAL4* in the visual system and EM data from NeuPrint. **(a)** The 1 mm thick side view of the three-color optic lobe image with nuclei (DAPI, blue), sparse labelling visual neurons (mCD8GFP, green) and photoreceptors R7 and R8 (GMR-RFP, glow). **(b)** A highlighted photoreceptor with R7 and R8 axons. **(c)** The sliced views of photoreceptor marked in (b) where (1) and (2) with two separate R7 and R8 axons; while (3) R8 axon terminal and (4) R7 axon terminal. **(d) and (e)** The selected volume for the three-color volume image for nuclei (cyan), photoreceptor (magenta) and a pair of segmented L3 neuron (blue) and photoreceptor (glow). **(f)** The spatial arrangement of R7, R8 and L3 neurons adapted from EM data, NeuPrint. **(g)** The orientation and spatial distribution of R8, R7, L3, and Dm9 neurons from NeuPrint. **(h)** The zoomed in view for the R8, R7, L3, and Dm9 axons at the L3 terminal. **(i)** The sliced EM image indicated in (h) showing R8, R7, L3, and Dm9 distributions. The scale bars in yellow font subjected to expansion factors, (b)-(e).

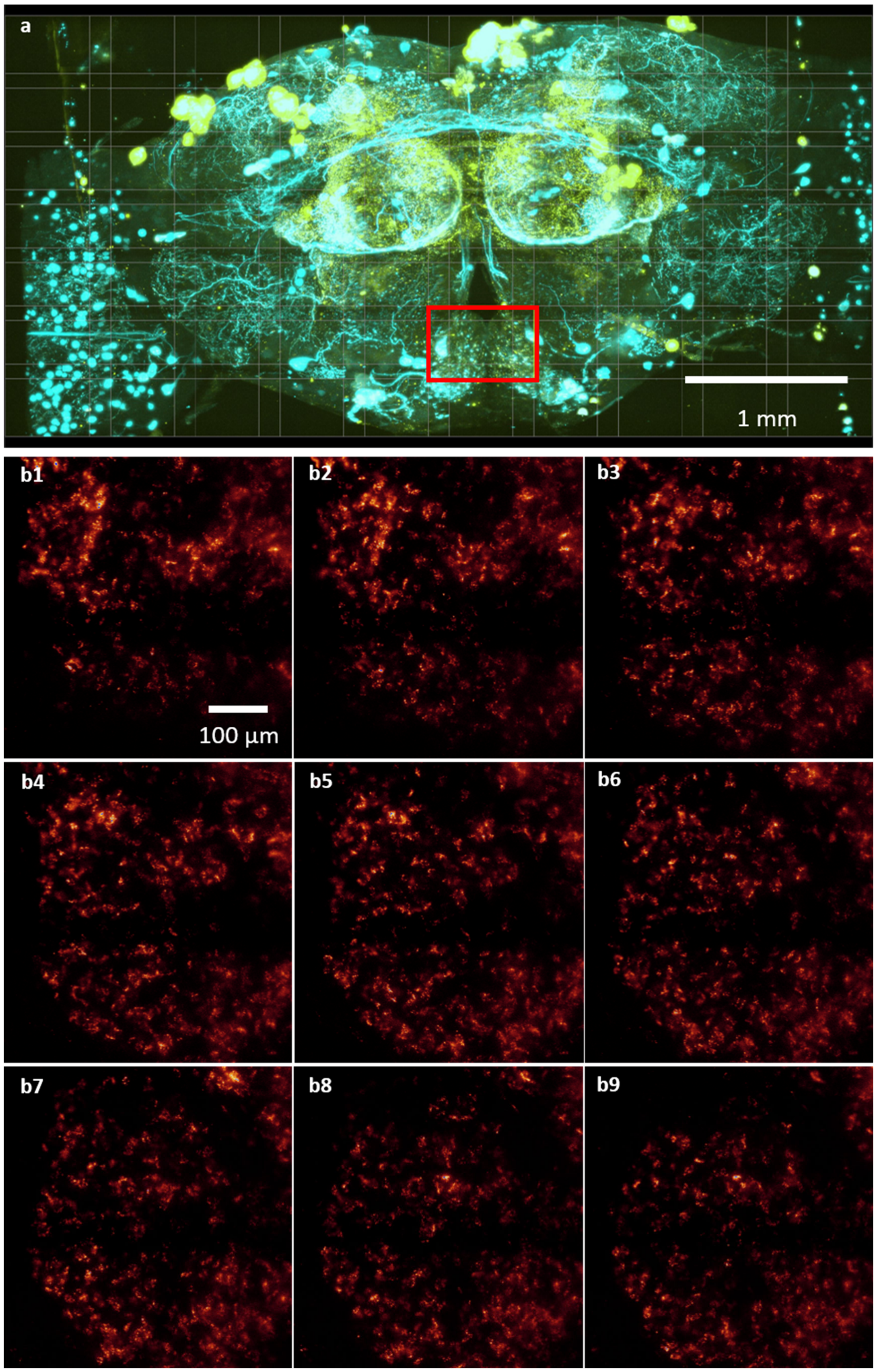
a
1 mm
b1
100 µm
b2
b3
b4
b5
b6
b7
b8
b9

Supplementary Figure 14. Lightsheet images of mitochondria and Brp^{Nc82} in the fly brain. **(a)** Tiled images of a UAS-mCherry.mito.OMM/+; TH-GAL4, 20XUAS-6XGFP/+ fly subjected to PKA-ExM and imaged by ΔBLX. Dopaminergic neurons (cyan) and mitochondria (yellow) are shown near the central complex area. The area marked by the red box is enlarged in Figure 7(a). **(b)** Slice views of Brp^{Nc82} labeling in the expanded asymmetrical body (AB) of a 3D volume rendering shown in blue in Fig. 7c, at a z interval of 5 μm.

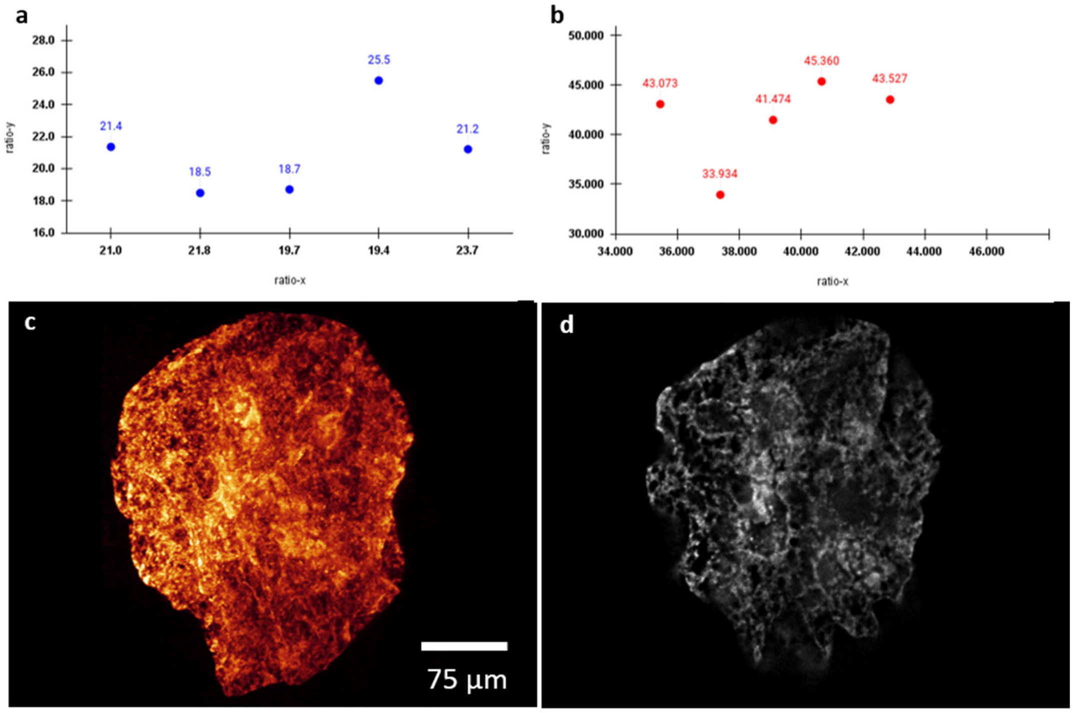

Supplementary Figure 15. The expansion ratio of *re*-KA and *re*-PKA ExM based on 100 µm spheroids generated from the HCT116 cell line in which nuclei have been stained by DAPI and imaged by ΔBLX. **(a)** Five trials of *re*-KA ExM treatment, resulting in a ~20x-plus expansion ratio. Expansion ratios calculated in two directions is 21.12 ± 1.75 and 21.05 ± 2.83 **(b)** Five trials of *re*-PKA ExM treatment, resulting in a ~40x-plus expansion ratio. Expansion ratios calculated in two directions is 39.09 ± 3.31 and 41.47 ± 5.12 **(c)** Raw 3D MIP image of one 330-µm interphase nucleus selected from Figure 9 (b) after *re*-PKA-ExM. **(d)** Single slice view of (c) showing the detailed structure of the nucleus.

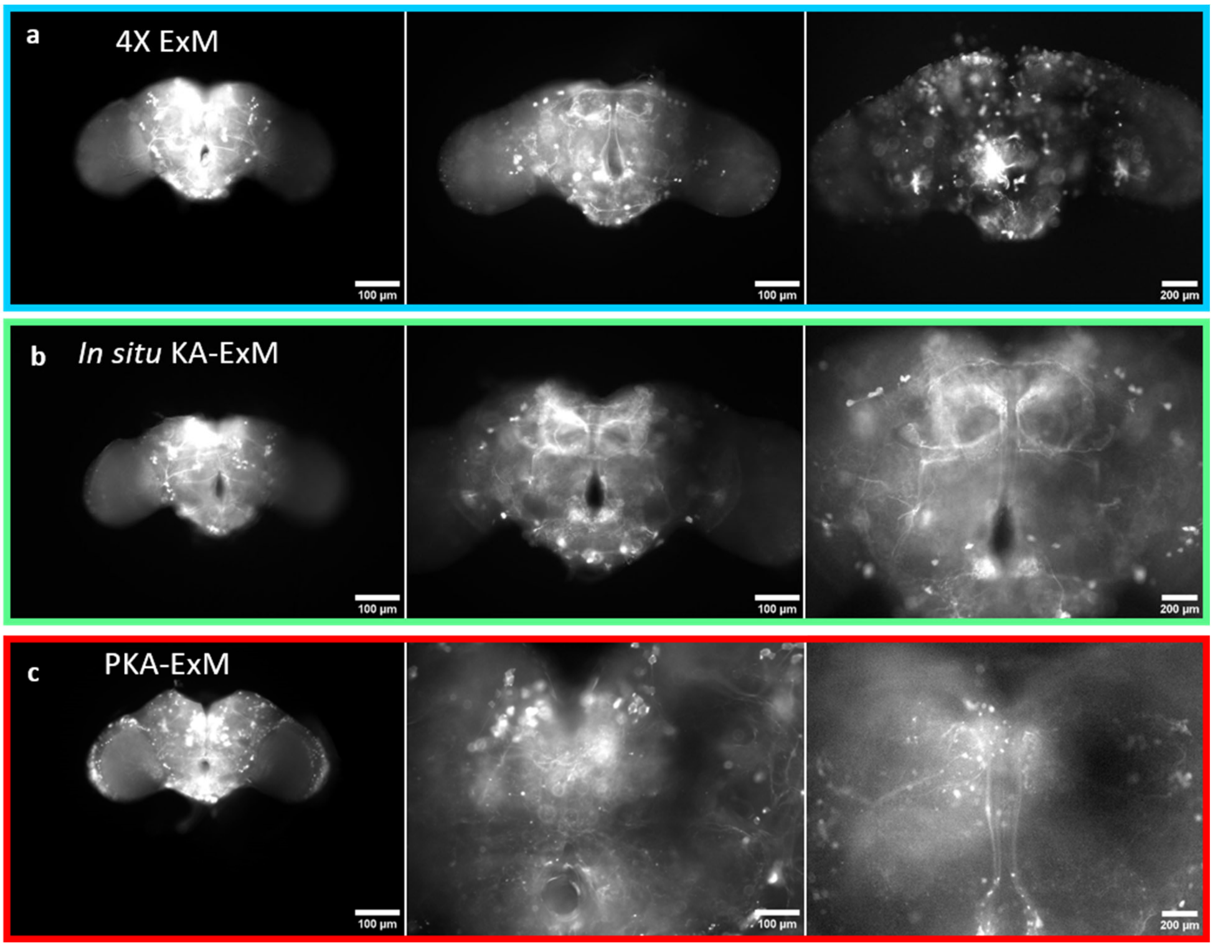

Supplementary Figure 16. The epi-fluorescence imaging for original (left), after digestion treatment (middle) and water expansion (right) on TH-GAL4, 20XUAS-6XGFP/+ *Drosophila* brain for **(a)** 4x ExM from Pro-ExM protocolprotocol in blue **(b)** 8x, *in situ* KA-ExM in green and **(c)**15x, PKA-ExM in red. Note that the scale bar 100 µm for original and after digestion buffer (DB); 200 µm for post-ExM. After DB treatment, expanded ratios measured at **1.3**, **1.8,** and **4**, respectively. After water expansion, final expanded ratios measured at **4**, **8**, and **15***,* respectively.

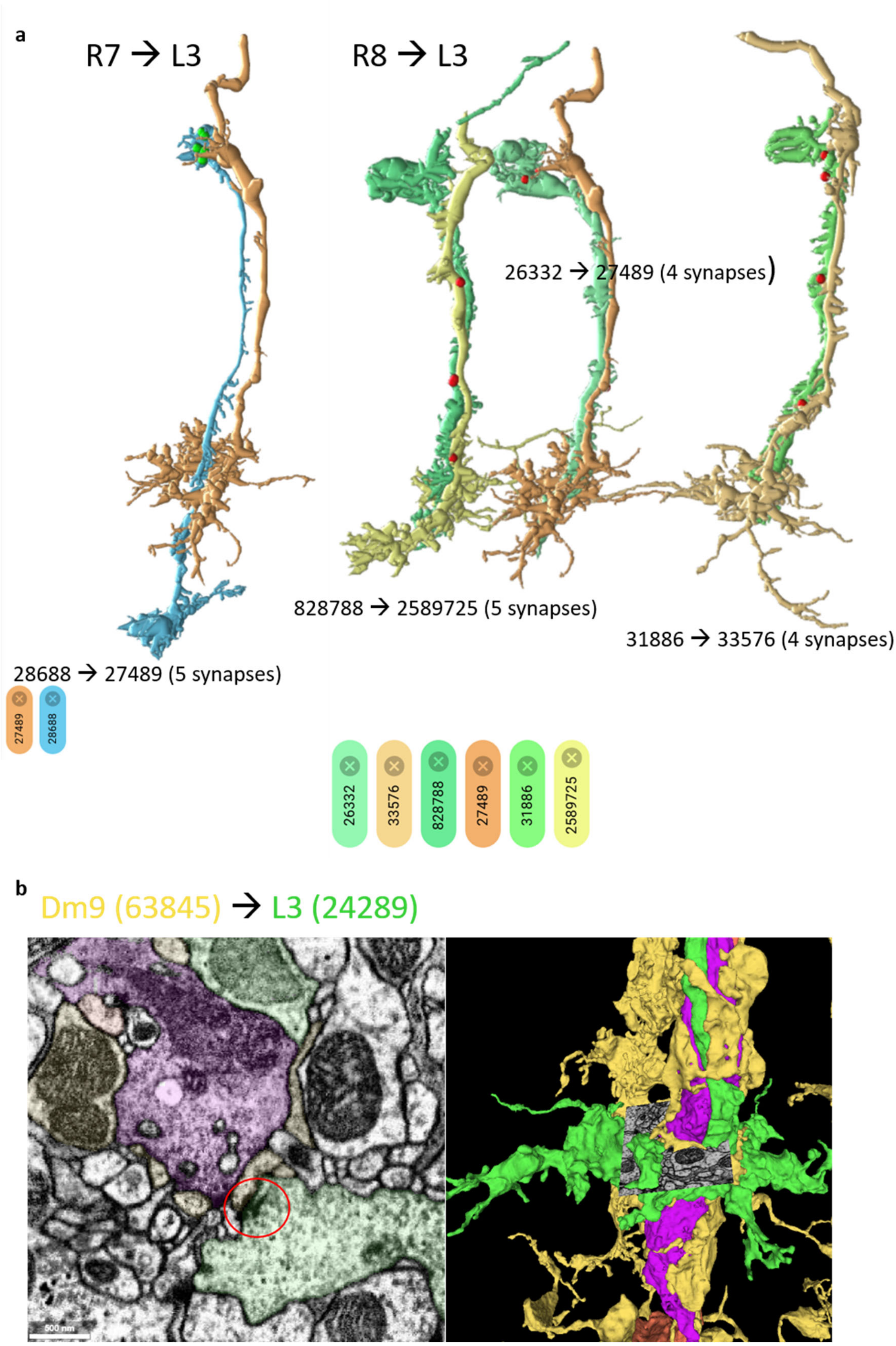
a
R7 → L3
R8 → L3
26332 → 27489 (4 synapses)
828788 → 2589725 (5 synapses)
31886 → 33576 (4 synapses)
28688 → 27489 (5 synapses)
27489
28688
26332
33576
828788
27489
31886
2589725
b
Dm9 (63845) → L3 (24289)
500 nm

Supplementary Figure 17. EM data from NeuPrint for R7, R8, L3, and Dm9 axons. **(a)** selected pairs of R7-L3 and R8-L3 for counting the synapse number from photoreceptors. ID from NeuPrint, R7 (28688) L3 (27489); R8 (828788) L3(2589725); R8(26332) L3(27489); R8(31886) L3(33576) **(b)** The pair of Dm9 (63845) and L3(24289) located near L3 terminal. The sliced EM image showing a T bar (synapse) structure between Dm9 and L3.

Supplementary Table 1. Summary of the various expansion protocols.

SAMPLE TYPE	SAMPLE SIZE	EXPANSION FACTOR	MONOMER COMPONENTS	SOURCE
BS-C-1 cell line<br>Mouse brain slice	100 or 150 μm (thickness)	16 to 22X	SA, AA, DHEBA (First expansion)<br>SA, AA, BIS (Second expansion)	Jae-Byum Chang et al., (2017), Nature Methods
Rat primary hippocampal neuron		10X	SA, DMAA	Sven Truckenbrodt et al., (2019) Nature protocols
Drosophila brain<br>Mouse cortex section<br>Human kidney glomerulus section	100 μm (thickness)<br>5 μm (thickness)	4X	SA, AA, BIS	Ruixuan Gao et al., (2019), Science
HeLa cell line<br>Mouse brain tissue	70 μm (thickness)	14 to 20X<br>23X	SA, AA, DHEBA (First expansion)<br>SA, AA, BIS (Second expansion)	Ons M'Saad et al. (2022), bioRxiv
U2OS cell line<br>Mouse brain tissue	100 μm (thickness)	8.8X	SA, AA, BIS	Hugo GJ Damstra et al., (2022), eLife
Drosophila brain		iterative 8X	SA, AA, BIS	Joshua L Lillvis et al., (2022), eLife
Drosophila brain		4.65X	SA, AA, BIS	Piero Sanfilippo et al., (2024), Neuron
Mouse primary somatosensory cortex	396 x 109 x 22 μm^3	15.44X	SA, AA, BIS (First expansion)<br>SA, AA, BIS (Second expansion)	Mojtaba R. Tavakoli et al., (2024), bioRxiv
Drosophila brain<br>Cos7 cell line<br>HCT116 tumor spheroids	500 x 300 x 200 μm^3<br>100 μm diameter	8 to 10X (KA-ExM)<br>~13 to 20X (re-KA)	KOH, Acrylic Acid (liquid), BIS (KA-ExM)<br>SA, AA, BIS (re-KA)	(*re*)-KA-ExM
Drosophila brain<br>Cos7 cell line<br>HCT117 tumor spheroids	500 x 300 x 200 μm3<br>100 μm diameter	10 to 15X (PKA-ExM)<br>>40X (re-PKA)	PKA, DMAA (PKA-ExM)<br>SA, AA, BIS (re-PKA)	(*re*)-PKA-ExM

AA: Acrylamide, BIS: N,N'-Methylenebisacrylamide, DHEBA: N,N′ -(1,2-dihydroxyethylene)bisacrylamide, SA: Sodium acrylate, DMAA: N,N-Dimethylacrylamide, PKA: potassium polyacrylate

Supplementary Table 2. The components of the axicon-based Bessel lightsheet microscope (ΔBLX).

COMPONENTS	MODEL
405 nm laser	Oxxius, LBX-405-300-CIR-PP
488 nm laser	MPB Communications, 2RU-VFL-P-300-488-B1R
560 nm laser	MPB Communications, 2RU-VFL-P-500-560-B1R
642 nm laser	MPB Communications, 2RU-VFL-P-500-642-B1R
acousto-optical tunable filter	AA Quanta Tech, AOTF AOTFnC-400.650-TN
Half wave plate	Bolder Vision Optik, BVO AHWP3
8 mm FL/12.24 mm dia	Thorlabs, C240TME-A
20 mm FL/12.5 mm dia	Edmund, 47-661
60 mm FL/25.4 mm dia	Thorlabs, AC254-060-A
200 mm FL/25.4 mm dia	Thorlabs, ACY254-200-A
250 mm FL/25.4 mm dia	ThorLabs, ACY254-250-A
Axicon	Thorlabs, AX2505-A
100 mm FL/25 mm dia	Edmund NT47-641
125 mm FL/25 mm dia	Edmund NT49-361
5 mm galvos	Cambridge Technology, 6215H
75 mm FL/25 mm dia	Edmund NT47-639
75 mm FL/25 mm dia	Edmund NT47-639
150 mm FL/25 mm dia	Edmund NT49-285
400 mm FL/75 mm dia	Edmund 88-595
a customized excitation objective	N.A. = 0.5, working distance = 12.8 mm, TIRI, Taiwan
detection objective	Olympus, XLPLN10XSVMP 10X, 0.6 NA, 8 mm WD
ultrasonic piezo motors	Physik Instrumente, PILine linear stage, U521
DC motor	Physik Instrumente, microtranslation stage, M122
voice coil stage	Physik Instrumente, Voice Coil PIFOC Focus, V308
sCMOS camera	Hamamatsu, Orca Flash 4.0 v2 sCMOS
350 mm FL/30 mm dia	OptoSigma DLB-30-350PM

Supplementary Table 3. Expanded *Drosophila* brain sample information and ΔBLX imaging conditions at excitation (NAout=0.17, NAin=0.16) and detection (NA=0.6) objectives.

SAMPLE	EXPANSION METHOD (M=ratio)	LABELING	SAMPLE VOLUME X × Y × Z (mm) Tiles(X × YxZ)	IMAGE PIXEL (X × Y × Z)/tile DATA SIZE (TB)	PIXEL RESOLUTION X × Y × Z (µm) (X/M x Y/M x Z/M)	IMAGING TIME/SPEED PER CHANNEL
**Figure 1d**	*In situ* KA	TH-GAL4, 20XUAS-6XGFP/+, DAPI staining	4.6 × 2.2 × 2.5	1408x2048x850	0.325 × 0.325 × 3	2 hrs for 2 colors
	M=8		12x5	0.5	(0.04x0.04x0.375)	1.8 mins /mm^3
	re-KA		7.5 × 2.9 × 4	1408x2048x1318	0.325 × 0.325 × 3	12 hrs for 2 colors
	M=13		20 × 8x1	2.3	(0.025x0.025x0.23)	1.8 mins /mm^3
**Figure 3b**	PKA	TH-GAL4, 20XUAS-6XGFP/+, DAPI staining	6.5 × 2.8 × 2.5	1408x2048x850	0.325 × 0.325 × 3	6 hours for 2 colors
	M=10		18 × 7x1	1.1	(0.03x0.03x0.3)	1.8 mins /mm^3
**Figure 4a**	PKA	TH-GAL4, 20XUAS-6XGFP/+, DAPI staining	6.8x3.4x2.5	1408x2048x514	0.325 × 0.325 × 5	1.6 hrs for 1 color
	M=12		19 × 6x1	0.315	(0.027x0.027x0.42)	1.1 mins /mm^3
	re-PKA		9.2x7.4x5.7 (half brain)	1408x2048x1226	0.325 × 0.325 × 5	14 hrs for 1 color
	M=32		23 × 18x1	2.67	(0.027x0.027x0.42)	1.1 mins /mm^3
**Figure 5a**	*re*-PKA	CD4-tdGFP	7.5x5.8x4.5	1408x2048x501	0.325 × 0.325 × 3	3 hrs for 1 color
	M= > 40		12 × 10x3	1	(0.008x0.008x0.075)	1.8 mins /mm^3
**Figure 7a**	*In situ* KA	mCD8-GFP, GMR-mRFP, DAPI staining	1.75x1x1	2048x2048x1001	0.104 × 0.104 × 1	6 hrs for 1 color
	M=10		20 × 10x1	2.4	(0.01x0.01x0.1)	34 mins /mm^3
**Figure 8a** (Supplementary 12a)	PKA	UAS-mCherry.mito.OMM/+; TH-GAL4, 20XUAS-6XGFP/+	5.5x2.65x2.4	1408x2048x501	0.325 × 0.325 × 3	4 hrs for 2 color
	M= 13		10 × 7x2	0.8	(0.027x0.027x0.23)	1.8 mins /mm^3
**Figure 8c**	*re*-PKA	BrpNc82-Cy3, immunostaining	7x6.3x3.9	1408x2048x301	0.325 × 0.325 × 5	6 hrs for 1 color
	M=40		13x17x3	1.1	(0.08x0.08x0.125)	1.1 mins /mm^3
**Figure 9b**	*re*-PKA	HCT spheroid, DAPI staining	4.5 × 4 × 3.5	1408x2048x1280	0.325 × 0.325 × 2.6	3 hours for 1 color
	M=40		8 × 11x1	0.6	(0.08x0.08x0.065)	1.8 mins /mm^3

Supplementary Table 4. PKA-ExM on Drosophila brain for pre- and post-expansion measurements.

SAMPLE	X (pre-PKA-ExM) (um)	Y (pre-PKA-ExM) (um)	X (post-PKA-ExM) (um)	Y (post-PKA-ExM) (um)	X-SCALE FACTOR	Y-SCALE FACTOR	X/Y RATIO
**1**	643	300	8575	4063	13.3	13.5	0.98
**2**	612	296	8194	4237	13.4	14.3	0.93
**3**	648	303	9326	3908	14.4	12.8	1.11
**4**	644	319	9978	5137	15.5	16	0.96
**5**	599	292	9288	5098	15.5	17.4	0.89
**6**	642	274	8726	4876	13.6	17.8	0.76
**7**	635	308	8581	4905	13.5	15.8	0.85

Supplementary Table 5. Comparisons of in situ KA-ExM and PKA-ExM and their 2nd expansions.

METHODS	MONOMER COMPONENTS	EXPANISON RATIO (M) / EXPERIMENTAL TIME (T, day)	SAMPLE HANDLING	2nd EXPANSION RATIO (m) / EXPERIMENTAL TIME (t, day)	Total mag. (Mxm) / TOTAL TIME (T+t, day)	SAMPLE HANDLING
in situ KA-ExM	KOH + Acrylic Acid (liquid) + MBA	8~10 / 3	EASY	re-KA-ExM 1.5~2 / 2	~13 / 5	EASY
PKA-ExM	PKA + DMAA	10~15 / 3	SLIGHTLY DIFFICULT (hold by tweezer)	re-PKA-ExM 3~4 / 2	>40 / 5	EASY

MBA: N,N'-Methylenebisacrylamide, DMAA: N,N-Dimethylacrylamide, PKA: potassium polyacrylate.

Supplementary Table 6. AFM measurements done in bio-AFM for the Young's modulus on 4xExM, in situ KA-ExM, and PKM-ExM hydrogels.（unit: MPa)

TRIAL	4X ExM	in situ KA-ExM（8x)	PKA-ExM（15x)
**1**	2.295	0.018	Not Applicable
**2**	2.721	0.018	
**3**	1.763	0.021	
**4**	4.322	0.025	
**5**	2.083	0.025	
**6**	4.932	0.025	
**7**	1.679	0.025	
**Avg.**	2.82	0.022	
**SD**	1.28	0.003	

Supplementary Table 7. Buffer components and chemical name abbreviations.

BUFFER NAME	COMPONENTS	STORAGE CONDITION	USE CONDITIONS
Fixation solution	4% Paraformaldehyle (PFA), 0.2% Glutaldehyde (GA)	4 ° C	40 min in 4% PFA, then 20 min in 0.2% GA, 4 ° C
Blocking buffer	10% Normal goat serum (NGS), 2% Triton X-100, 0.02% Sodium azide in 1X PBS	4 ° C	1hr, 37 ° C
Antibody dilution buffer	1% NGS, 0.25% Triton X-100, 0.02% Sodium azide in 1X PBS	4 ° C	1-3 days, RT
Antibody wash buffer	1% Triton X-100 in 1X PBS	4 ° C	15 min, x3 times, RT
Anchoring solution	10mM Methacrylic acid N-hydroxy succinimidyl ester (MA-NHS) in 1X PBS	4 ° C	Overnight, 4 ° C
KA monomer solution	18.7% (w/v) Potassium hydroxide (KOH), 23.7% (v/v) Acrylic acid (AA-liquid), 0.03% (w/v)N,N'-methylenebisacrylamide (MBA) in ddH2O	-20 ° C	For gelation, 2hrs, 37 ° C
PKA monomer solution	21.8% (w/v) Potassium polyacrylate (PKA), 31.3% (v/v) Dimethylacrylamide (DMAA), in ddH$_2$O	-20 ° C	For gelation, 2hrs, 37 ° C
Gelation initiator	0.0036 g/mL Potassium peroxydisulfate (KPS), 0.4% Tetramethylethylenediamine (TEMED)	-20 ° C for KPS, RT for TEMED	For gelation, 2hrs, 37 ° C
Digestion buffer	50 mM Tris pH 8.0, 800 mM guanidine HCl, 2 mM CaCl2, and 0.5% (v/v) Triton X-100 in ddH$_2$O, with freshly added proteinase K diluted 1:100	-20 ° C for Proteinase K, RT for others	Overnight, RT
Re-embedding wash buffer	13.75% Acrylamide (AA), 0.038% MBA, 0.03% ammonium persulfate (APS), 0.03% TEMED, and 5 mM Tris pH 8.0 in ddH2O	Freshly prepared	10 min, x2 times, then 1 hr, RT
Re-embedding gelling solution	13.75% AA, 0.038% MBA, 0.05% APS, 0.05% TEMED, and 5 mM Tris pH 8.0 in ddH2O	Freshly prepared	2 hrs, 45 ° C
Second gelling solution	2 M NaCl, 8.6% (w/w) sodium acrylate (SA), 2.5% (w/w) AA, 0.15% (w/w) MBA in 1X PBS, with freshly added 0.03% APS and 0.03% TEMED	Freshly prepared	1 hr, 45 ° C

Supplementary Table 8. Chemical reagents list.

REAGENT	SOURCE	IDENTIFIER
4-hydroxy-2,2,6,6-tetramethylpiperidin-1-oxyl (4-hydroxy-TEMPO)	Sigma-Aldrich	176141-1G
Acrylamide (AA)	Sigma-Aldrich	A8887-100G
Acryloyl-X (AcX)	ThermoFisher	A20770
Ammonium persulfate (APS)	Sigma-Aldrich	A9164-100G
Calcium chloride	J.T. Backer	1311-01
Glutaraldehyde 8% aqueous solution	Electron Microscopy Sciences	16020
Glutaraldehyde solution, 25%	Sigma-Aldrich	G6257-100ML
Guanidine HCl	Sigma-Aldrich	G4505-25G
Methacrylic Acid N-hydroxy Succinimidyl Ester (MA-NHS)	Sigma-Aldrich	730300-1G
N,N,N',N'-Tetramethylethylene-1,2-diamine (TEMED)	Sigma-Aldrich	T7024-25ML
N,N-Dimethlacrylamide (DMAA)	Sigma-Aldrich	274135-500ML
N,N'-Methylenebisacrylamide (MBA)	Sigma-Aldrich	M7256-25G
Paraformaldehyde (PFA)	Sigma-Aldrich	16005-1KG
PBS (10x)	UniRegion Bio-Tech	UR-PBS001-1L
Potassium acrylate	ChemScene	CS-0196459
Potassium hydroxide	Sigma-Aldrich	30603-1KG
Potassium persulfate(KPS)	J.T. Backer	3238-01
Proteinase K Solution (RNA grade) 20mg/ml	ThermoFisher	P/N 100005393
Sodium acrylate (SA)	Sigma-Aldrich	408220-100G
Sodium chloride (NaCl)	J.T. Backer	3624-69
Tris (1 M), pH 8.0, RNase-free	ThermoFisher	AM9856
Triton X-100	Sigma-Aldrich	X100-100ML
DAPI (1 mg/ml)	ThermoFisher	62248
DAPI Fluoromount-G®	Southernbiotech	#0100-20
Sodium azide	Sigma-Aldrich	S2002-100G
NGS(normal goat serum)	Gibco	16210-064-100ml
Anti-GFP (Primary antibodies/Rabbit / Polyclonal Antibody)	ThermoFisher	A-11122
Mouse anti-Brp	DSHB	nc82
Goat anti-Mouse conjugate Cy3	Jackson ImmunoResearch	115-165-166
Goat anti-Rabbit IgG (H+L) Secondary Antibody, Biotin	ThermoFisher	65-6140
Streptavidin, Alexa Fluor™ 635 conjugate (SA635)	ThermoFisher	S32364
