# Supplementary material for: Rapid lightsheet fluorescence imaging of whole Drosophila brains at nanoscale resolution by potassium acrylate-based expansion microscopy
Source: Nat Commun. 2024 Dec 30;15:10911. doi: 10.1038/s41467-024-55305-8 (PMC11685761; doi:10.1038/s41467-024-55305-8)
Supplement: Supplementary file 2 — Reporting Summary [file 41467_2024_55305_MOESM2_ESM.pdf]

nature portfolio

Corresponding author(s): Bi-Chang Chen

Last updated by author(s): Nov 22, 2024

Reporting Summary

Nature Portfolio wishes to improve the reproducibility of the work that we publish. This form provides structure for consistency and transparency in reporting. For further information on Nature Portfolio policies, see our Editorial Policies and the Editorial Policy Checklist.

Statistics

For all statistical analyses, confirm that the following items are present in the figure legend, table legend, main text, or Methods section.

n/a	Confirmed	
☐	☒	The exact sample size (*n*) for each experimental group/condition, given as a discrete number and unit of measurement
☐	☒	A statement on whether measurements were taken from distinct samples or whether the same sample was measured repeatedly
☒	☐	The statistical test(s) used AND whether they are one- or two-sided<br>*Only common tests should be described solely by name; describe more complex techniques in the Methods section.*
☒	☐	A description of all covariates tested
☒	☐	A description of any assumptions or corrections, such as tests of normality and adjustment for multiple comparisons
☐	☒	A full description of the statistical parameters including central tendency (e.g. means) or other basic estimates (e.g. regression coefficient) AND variation (e.g. standard deviation) or associated estimates of uncertainty (e.g. confidence intervals)
☒	☐	For null hypothesis testing, the test statistic (e.g. *F*, *t*, *r*) with confidence intervals, effect sizes, degrees of freedom and *P* value noted<br>*Give P values as exact values whenever suitable.*
☒	☐	For Bayesian analysis, information on the choice of priors and Markov chain Monte Carlo settings
☒	☐	For hierarchical and complex designs, identification of the appropriate level for tests and full reporting of outcomes
☒	☐	Estimates of effect sizes (e.g. Cohen's *d*, Pearson's *r*), indicating how they were calculated

Our web collection on statistics for biologists contains articles on many of the points above.

Software and code

Policy information about availability of computer code

Data collection	labview software,
Data analysis	Image J, Amira 3D 2024.1, Imaris 9.5, Autodesk Inventor,ITK-SNAP, Huygens Software

For manuscripts utilizing custom algorithms or software that are central to the research but not yet described in published literature, software must be made available to editors and reviewers. We strongly encourage code deposition in a community repository (e.g. GitHub). See the Nature Portfolio guidelines for submitting code & software for further information.

Data

Policy information about availability of data

All manuscripts must include a data availability statement. This statement should provide the following information, where applicable:

- Accession codes, unique identifiers, or web links for publicly available datasets
- A description of any restrictions on data availability
- For clinical datasets or third party data, please ensure that the statement adheres to our policy

Source data are provided with this paper. Due to the data size limit, only the binning lightsheet data or partial raw image data generated in this study have been deposited in the BioStudies database53 under accession code S-BIAD1488 [https://www.ebi.ac.uk/biostudies/bioimages/studies/S-BIAD1488]. Requests for the raw image data should be addressed to B.C.C. (chenb10@gate.sinica.edu.tw).

Research involving human participants, their data, or biological material

Policy information about studies with human participants or human data. See also policy information about sex, gender (identity/presentation), and sexual orientation and race, ethnicity and racism.

Reporting on sex and gender	NA
Reporting on race, ethnicity, or other socially relevant groupings	NA
Population characteristics	NA
Recruitment	NA
Ethics oversight	NA

Note that full information on the approval of the study protocol must also be provided in the manuscript.

Field-specific reporting

Please select the one below that is the best fit for your research. If you are not sure, read the appropriate sections before making your selection.

- [x] Life sciences
- [ ] Behavioural & social sciences
- [ ] Ecological, evolutionary & environmental sciences

For a reference copy of the document with all sections, see nature.com/documents/nr-reporting-summary-flat.pdf

Life sciences study design

All studies must disclose on these points even when the disclosure is negative.

Sample size	we are providing the protocol and imaging platform for the new type of ExM and testing the related parameters for chemical reagents and all the results are provided in the supporting materials.
Data exclusions	no data were excluded from the analyses
Replication	We measured the pre/post expansion sample size, given that the chemicals such as monomer or initiator are stored in good condition. The expansion ratio we have are expected. Sometimes the expansion process not run well is either because the sample dissection is not good or DMAA decomposed
Randomization	we used dissected adult fly brains or spheroid (HCT) as subjects for the KA-ExM lightsheet imaging. We care pre- and post expansion results on the individual same subject. no randomization is need here.
Blinding	no blinding to our study

Reporting for specific materials, systems and methods

We require information from authors about some types of materials, experimental systems and methods used in many studies. Here, indicate whether each material, system or method listed is relevant to your study. If you are not sure if a list item applies to your research, read the appropriate section before selecting a response.

Materials & experimental systems

n/a	Involved in the study	
[ ]	[x]	Antibodies
[ ]	[x]	Eukaryotic cell lines
[x]	[ ]	Palaeontology and archaeology
[ ]	[x]	Animals and other organisms
[x]	[ ]	Clinical data
[x]	[ ]	Dual use research of concern
[x]	[ ]	Plants

Methods

n/a	Involved in the study	
[x]	[ ]	ChIP-seq
[x]	[ ]	Flow cytometry
[x]	[ ]	MRI-based neuroimaging

Antibodies

Antibodies used	1. GFP Polyclonal Antibody, ThermoFisher A-11122, LOT Number: 2659306, 1:100 dilution in Antibody dilution buffer (Supplementary Table 7);<br>2. Brp Monoclonal Antibody, DSHB nc82, LOT Number: 4/16/20-53µg/mL, 1:50 in Antibody dilution buffer (Supplementary Table 7);

3. Cy™3 AffiniPure™ Goat Anti-Mouse IgG (H+L), Jackson ImmunoResearch 115-165-166, LOT Number: 124102, 1:100 in Antibody dilution buffer (Supplementary Table 7);
4. Goat anti-Rabbit IgG (H+L) Secondary Antibody, Biotin, ThermoFisher 65-6140, LOT Number: VH310381, 1:100 dilution in Antibody dilution buffer (Supplementary Table 7).

Validation

1. GFP antibody (from manufacturer datasheet):
Product Details
Size: 100 µL
Species Reactivity: Tag
Published Species: Tag
Host/Isotype: Rabbit / IgG
Class: Polyclonal
Type: Antibody
Conjugate: Unconjugated
Immunogen: The GFP was isolated directly from the jellyfish Aequorea victoria.
Form: Liquid
Concentration: 2 mg/mL
Purification: IgG fraction
Storage buffer: PBS, pH 7.2
Contains: 5mM sodium azide
Storage conditions: 4° C
RRID: AB_221569
Applications
Western Blot (WB) ,Immunohistochemistry (IHC), Immunohistochemistry (Paraffin) (IHC (P)), Immunohistochemistry (Frozen) (IHC (F)), Immunohistochemistry - Free Floating (IHC (Free)), Immunocytochemistry (ICC/IF), Flow Cytometry (Flow), ELISA (ELISA), Immunoprecipitation (IP), ChIP assay (ChIP), Neutralization (Neu), Functional Assay (Functional), in situ PLA (PLA), Immunomicroscopy (IM), Inhibition Assays (IA), Gel Shift (GS), RNA Immunoprecipitation (RIP), Affinity Purification (AP), In vitro Assay (IV).
2. Brp antibody (from manufacturer datasheet):
Clone ID/Product Name: nc82
Available to For-Profits: Yes
Gene Symbol: Brp
Ab Isotype: MIgG1, kappa light chain
Antibody Registry ID: AB_2314866
Uniprot ID: A1Z7V1
Entrez Gene ID: 35977
Clonality: Monoclonal
Immunogen: Head homogenate
Myeloma Strain: P3X63Ag8.653
Epitope Mapped: Yes
Antigen Name: Bruchpilot
Epitope Location or Sequence: C-terminal aa 1227-1740
Deposit Date: 6/10/2005
Antigen Molecular Weight: 201.6 kDa
Depositor: Buchner, E.
Depositor Institution: Universitaetsklinikim Wuerzburg
Antigen Species: Drosophila
Depositor Notes: This antibody specifically labels presynaptic active zones.
Host Species: mouse
Hybridoma Cells Available (Non-Profit): Yes
Confirmed Species Reactivity: Aedes aegypti, Drosophila, Mosquito
Recommended Applications: Immunofluorescence, Immunohistochemistry, Western Blot

3. Cy™3 AffiniPure™ Goat Anti-Mouse IgG (H+L) (from manufacturer datasheet):
Target: Mouse
Host: Goat
Antibody Format: Whole IgG
Specificity: IgG (H+L)
Minimal Cross Reactivity: Human, Bovine, Horse, Rabbit, Rat Serum Proteins
Conjugate: Cyanine Cy™3
Product Category: Whole IgG Affinity-Purified Antibodies
Clonality: Polyclonal
RRID: AB_2338692
Antibody Specificity: Based on immunoelectrophoresis and/or ELISA, the antibody reacts with whole molecule mouse IgG. It also reacts with the light chains of other mouse immunoglobulins. No antibody was detected against non-immunoglobulin serum proteins. The antibody has been tested by ELISA and/or solid-phase adsorbed to ensure minimal cross-reaction with rat, human, bovine, horse, and rabbit serum proteins, but it may cross-react with immunoglobulins from other species.

4. Goat anti-Rabbit IgG (H+L) Secondary Antibody, Biotin (from manufacturer datasheet):

Product Details
Size: 2 mL
Species Reactivity: Rabbit
Host/Isotype: Goat / IgG
Class: Polyclonal
Type: Secondary Antibody
Conjugate: Biotin
Immunogen: Gamma Immunoglobin
Form: Liquid
Concentration: 0.6 mg/mL
Purification Affinity: chromatography
Storage buffer: PBS, pH 7.4, with 1% BSA, 40% glycerol
Contains: 0.1% sodium azide
Storage conditions: 4° C
RRID: AB_2533969
Applications
Western Blot (WB), Immunohistochemistry (IHC), Immunohistochemistry (Paraffin) (IHC (P)), ELISA (ELISA), Immunoprecipitation (IP).

Eukaryotic cell lines

Policy information about cell lines and Sex and Gender in Research

Cell line source(s)	Human colorectal cells HCT 116 (BCRC 60349, Bioresource collection and Research Center, Taiwan)
Authentication	HCT 116 cell line was isolated from the colon of an adult male with colon cancer. It has a mutation in codon 13 of the ras proto-oncogene and can be used as a positive control for PCR assays of mutation in this codon, HCT 116 (ATCC CCL-247)
Mycoplasma contamination	no mycoplasma contamination
Commonly misidentified lines (See ICLAC register)	NA

Animals and other research organisms

Policy information about studies involving animals; ARRIVE guidelines recommended for reporting animal research, and Sex and Gender in Research

Laboratory animals	Drosophila melanogaster brain, Fly stocks were raised on cornmeal food, and maintained at a temperature of 25 °C and 70% relative humidity under a 12-h light/dark cycle. The following fly lines were used in the current study: TH-GAL4 (8848, BDSC); 20XUAS-6XGFP@attP2 (52262, BDSC); UAS-mCherry.mito.OMM (66532, BDSC); 13xLexAop2-CD4-tdGFP (77136, BDSC); OrtC1-3-GAL4, GMR-myr-mRFP (7121, BDSC), and Tm5a split-LexA (from T.Y.Lin and C.H.Lee). The following fly genotypes were generated based on the aforementioned fly strains: TH-GAL4>20XUAS-6XGFP, UAS-mCherry.mito.OMM for labeling mitochondria in dopaminergic neurons; Tm5a split-LexA>13xLexAop2-CD4-tdGFP to sparsely label Tm5a neurons; and a L3 single cell MARCM clone generated by OrtC1-3-GAL4 in the visual system.
Wild animals	NA
Reporting on sex	NA
Field-collected samples	NA
Ethics oversight	ethical approval was not required

Note that full information on the approval of the study protocol must also be provided in the manuscript.

Plants

Seed stocks	NA
Novel plant genotypes	NA
Authentication	NA
