# Supplementary material for: Rapid lightsheet fluorescence imaging of whole Drosophila brains at nanoscale resolution by potassium acrylate-based expansion microscopy
Source: Nat Commun. 2024 Dec 30;15:10911. doi: 10.1038/s41467-024-55305-8 (PMC11685761; doi:10.1038/s41467-024-55305-8)
Supplement: Supplementary file 3 — Description of Additional Supplementary Files [file 41467_2024_55305_MOESM3_ESM.pdf]

Supplementary Movie 1. The *in situ* KA-hydrogel in 5-mm size on the epi-fluorescence microscope with different views by rotating and good for vibratome sectioning into a thin slice, the hydrogel is dyed with red color for visibility. Associated with Figure 1.

Supplementary Movie 2. 3D renderings of the *in situ* KA-ExM on the TH-GAL4, 20XUAS-6XGFP/+ *Drosophila melanogaster* brain, acquired by ΔBLX. The resulted *8x* expansion fly brain with nuclei (purple color) and dopaminergic neurons (glow color) with z interval of 3 µm, total ~2.0 mm thick. The zoomed-in view for the central complex region for the details imaged by NA=0.6 objective lens. Associated with Figure 1.

Supplementary Movie 3. 3D renderings of the PKA-ExM on the TH-GAL4, 20XUAS-6XGFP/+ *Drosophila melanogaster* brain, acquired by ΔBLX. The resulted *10x* expansion fly brain with nuclei (purple color) and dopaminergic neurons (glow color) with z interval of 3 µm, total ~2.3 mm thick. The one tile marked in cyan, showing the raw images for nuclei (red) and dopaminergic neurons (green color) for zoomed-in view for button-like structures and nano-sized neuron fibers near the central complex region in fly brain, imaged by NA=0.6 objective lens. Associated with Figure 3.

Supplementary Movie 4. 3D renderings of the *re*-PKA-ExM on the *Drosophila melanogaster* Tm5a visual neurons, acquired by ΔBLX with z interval of 5 µm to cover 4 mm thick sample. The segmented ones in yellow overlaid with raw signals from expansion optical lobe with optical slice view for the detailed structures, imaged by NA=0.6 objective lens Associated with Figure 5.

Supplementary Movie 5. 3D renderings of the *re*-PKA-ExM on the *Drosophila melanogaster* Tm5a visual neurons, acquired by ΔBLX with z interval of 5 µm to cover 4 mm thick sample. A group of densely packed Tm5a neurons could be segmented, where ~15 neurons are colored and skeletonized as shown in Supplementary Fig. 12. Associated with Figure 5.
